# Crosstalk Between PD-1/PD-L1 Blockade and Its Combinatorial Therapies in Tumor Immune Microenvironment: A Focus on HNSCC

**DOI:** 10.3389/fonc.2018.00532

**Published:** 2018-11-21

**Authors:** Weimin Lin, Miao Chen, Le Hong, Hang Zhao, Qianming Chen

**Affiliations:** State Key Laboratory of Oral Diseases, West China Hospital of Stomatology, Sichuan University, Chengdu, China

**Keywords:** cancer immunotherapy, combined therapy, PD-1, PD-L1, head and neck squamous cell carcinoma

## Abstract

Head and neck squamous cell carcinoma (HNSCC) is the sixth most common malignancy worldwide with a poor prognosis and high mortality. More than two-thirds of HNSCC patients still have no effective control of clinical progression, and the five-year survival rate is < 50%. Moreover, patients with platinum-refractory HNSCC have a median survival of < 6 months. The significant toxicity and low survival rates of current treatment strategies highlight the necessity for new treatment modalities. Recently, a large number of studies have demonstrated that programmed cell death protein-1 (PD-1) and its ligand, programmed cell death protein ligand-1 (PD-L1) play an essential role in tumor initiation and progression. PD-1/PD-L1 blockade has shown a desired and long-lasting therapeutic effect in the treatment of HNSCC and other malignancies. However, only a small number of patients with HNSCC can benefit from PD-1/PD-L1 blockade monotherapy, while the majority of patients do not respond. To overcome the unsatisfactory therapeutic effect of PD-1/PD-L1 blockade monotherapy, combining other treatment options for HNSCC (including chemotherapy, radiotherapy, targeted therapy, and immunotherapy) in the treatment scheme has become a commonly used strategy. Herein, the potential mechanisms underlying the crosstalk between PD-1/PD-L1 blockade and its combinatorial therapies for HNSCC were reviewed, and it is hoped that the improved understanding of the crosstalk process would provide further ideas for the design of a combinatorial regimen with a higher efficiency and response rate for the treatment of HNSCC and other malignancies.

## Introduction

Head and neck squamous cell carcinoma (HNSCC) includes squamous cell carcinomas that occurs in the nasopharynx, oropharynx, hypopharynx, and throat. Its incidence rate is ranked 6th among common malignancies worldwide (1). Tumor recurrence and metastasis occurs in more than 50% of patients with HNSCC within three years. Only few choices exist for the treatment of recurrent or metastatic (R/M) HNSCC, leading to its poor prognosis. In addition, a higher proportion of patients with R/M HNSCC often have tumor-related symptoms, including pain, hemorrhage, respiratory, and nutritional disorders, which seriously affect the quality of life of patients and the choice of follow-up treatment (2). In 2016, the US Food and Drug Administration (FDA) approved the PD-1-targeted monoclonal antibodies (mAbs), pembrolizumab, and nivolumab, for the treatment of R/M HNSCC. The new edition of the National Comprehensive Cancer Network incorporated these two drugs for the treatment of HNSCC (3). Compared with traditional therapies, the emerging PD-1 blockade immunotherapy exhibited an encouraging therapeutic efficacy for patients with advanced HNSCC (4).

Due to the inherent genetic inheritance and high somatic mutation in HNSCC, alterations in the protein repertoire can be introduced, which usually result in the expression of neoantigens that can be recognized by T cells and tumor cell clearance by immune surveillance system (5, 6). In addition, in addition to the neoantigen pathway generated by cancer genome mutations, HNSCC associated with HPV infection can also be detected by T cells due to the viral antigen expression, thereby eliciting an anti-tumor immune response (7). Similarly, recent studies have revealed that HNSCC exhibits an enriched tumor immune landscape, and when HNSCC showed a clinical progression, the immune response elicited by the strong immunogenicity of HNSCC is usually suppressed. Immunosuppressive cells, such as bone marrow-derived suppressor cells (MDSCs), M2-type tumor-associated macrophages (TAMs), and regulatory T cells (Tregs) can build an immunosuppressive state in HNSCC tumor microenvironment (TME). At the same time, with the introduction of the concept of immune checkpoints, a series of inhibitory immune checkpoints including PD-1, CTLA-4, TIM-3, IDO, KIR, and TIGIT, have been proved to be involved in the construction of HNSCC immunosuppressive microenvironment (8). The blockade of immune checkpoint PD-1/PD-L1 could reduce the population and activity of MDSCs and Tregs (9), and restore the cytotoxicity of T cells and NK cells (10), ultimately relieving the tumor immune escape and inhibiting tumor growth.

A high expression of PD-L1 was commonly observed in HNSCC cells (40–70%) (11, 12), coinciding with the upregulation of PD-1 on the majority of CD8^+^ tumor-infiltrating lymphocytes (TILs) (11). Yu et al. found that PD-1 expression increased in samples from patients with HNSCC, in comparison with normal oral mucosa samples (13), which implied that the PD-1 blockade should be more effective at boosting the antitumor immune response in HNSCC. However, data from recent clinical trials revealed a modest overall response rate (ORR) to the PD-1 blockade of less than 20% (4, 14), and a lack of dramatic responses in most patients (15) when compared with the more impressive ORRs of up to 57% in other advanced/pretreated indications, such as non-small cell lung cancer and melanoma (16, 17). Thus, only a small number of patients with HNSCC can benefit from PD-1/PD-L1 blockade therapy.

**Figure 1:**
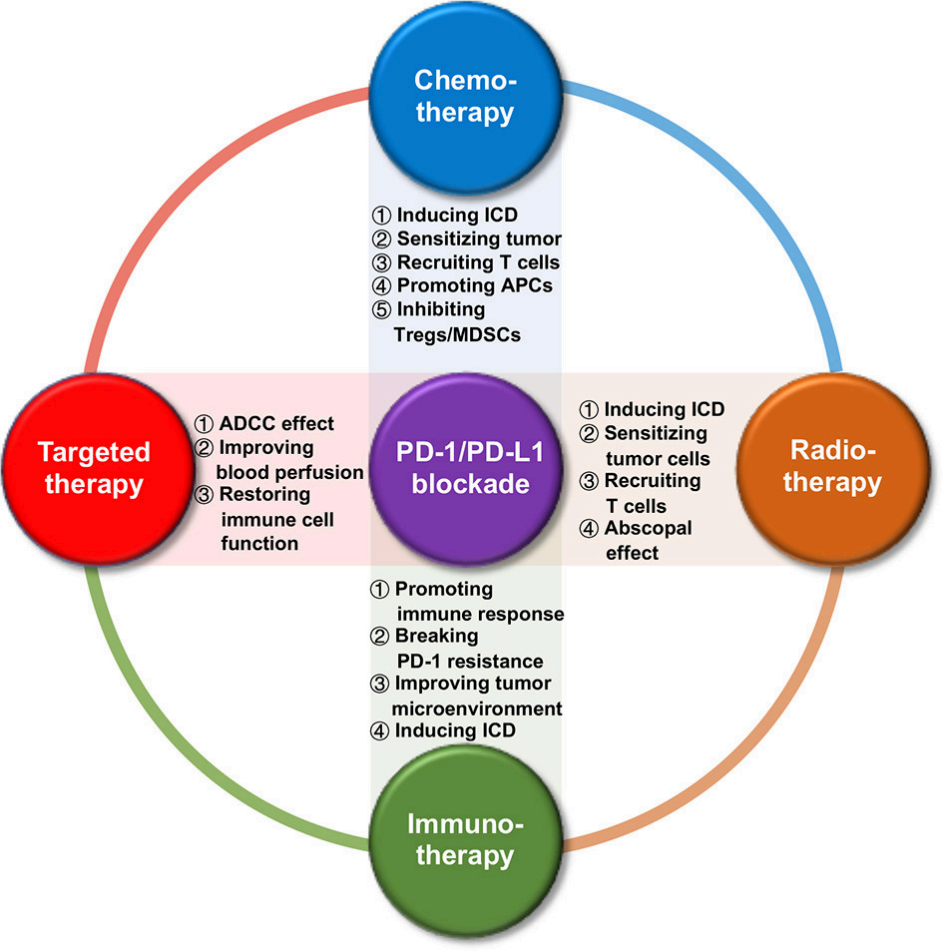
Synergistic effects between PD-1/PD-L1 blockade and its combinatorial therapies.

A growing number of preclinical and clinical trials have confirmed that traditional cancer therapies can promote the release of tumor-associated antigens (TAAs), increase antigen presentation, as well as enhance the expression of PD-1/PD-L1 axis. In the treatment of tumors with PD-1/PD-L1 blockade, combination with other therapies can provoke the antitumor immune response, which helps to improve the efficacy of PD-1/PD-L1 blockade. Thus, combinatorial immunotherapy represents a promising approach to boost antitumor activity in HNSCC as well as other malignancies. Therefore, it is especially important to understand the dynamic changes in the TME after standard treatment in order to design a rational combination treatment plan involving PD-1/PD-L1 blockade (18). In this review, we summarized its combination with other current treatment options for HNSCC (including chemotherapy, radiotherapy, targeted therapy, and immunotherapy) from their crosstalk in the TME (Figure 1) to the ongoing clinical trials (Table 1), in order to better understand the mechanism of interaction between PD-1/PD-L1 blockade and other cancer therapies, and to provide further ideas for the design of combinatorial regimen with a higher efficiency and response rate in HNSCC and other malignancies.

**Table 1:** Ongoing clinical trials (registered in ClinicalTrials.gov) involving combinatorial PD-1/PD-L1 blockade therapy in recurrent/metastatic head and neck squamous cell carcinoma as of June 2018.

**Clinical Trials. gov identifier**	**Mechanisms of action**	**Regimen (treatment arms)**	**Phase**	**Estimated enrolment**	**Primary completion date**
NCT03085719	Anti PD-1 Radiotherapy	Pembrolizumab + Radiation	2	26	2020/10/31
NCT03317327	Anti PD-1 Radiotherapy	Nivolumab + Radiation	1,2	20	2023/11/2
NCT02289209	Anti-PD-1 Radiotherapy	Pembrolizumab + Reirradiation	2	48	2018/12/1
NCT03313804	Anti-PD-1 Radiotherapy	Nivolumab or Pembrolizumab or Atezolizumab + Radiation	2	57	2018/6/30
NCT03058289	Anti-PD-1 Chemotherapy	A: INT230-6 (Cisplatin + Vinblastine + Cell Permeation Enhancer) B: Anti-PD-1 + INT230-6	1,2	60	2019/7/1
NCT02358031	Anti-PD-1 Chemotherapy	A: Pembrolizumab B: Pembrolizumab + Cisplatin+5-fluorouracil C: Cetuximab + Cisplatin + 5-fluorouracil	3	825	2018/12/31
NCT02710396	Anti-PD-1 Chemotherapy	A: Pembrolizumab B: Pembrolizumab + Carboplatin + Nab-paclitaxel C: Pembrolizumab + Carboplatin + Pemetrexed	2	90	2019/3/31
NCT02759575	Anti-PD-1 Chemotherapy Radiotherapy	Pembrolizumab + Cisplatin + Radiation	1,2	47	2020/1/1
NCT03040999	Anti-PD-1 Chemotherapy Radiotherapy	A: Pembrolizumab + Cisplatin + Radiation B: Placebo + Cisplatin + Radiation	3	780	2021/4/16
NCT02819752	Anti-PD-1 Chemotherapy Radiotherapy	Pembrolizumab + Cisplatin + Radiation	1	36	2018/3/1
NCT03082534	Anti-PD-1 Anti-EGFR	Pembrolizumab + Cetuximab	2	83	2019/5/27
NCT02646748	Anti-PD-1 JAK inhibitor PI3K-δ	A: Pembrolizumab + Itacitinib(JAK Inhibitor) B: Pembrolizumab + INCB050465(PI3K-δ Inhibitor)	1	237	2019/6/1
NCT03532737	Anti-PD-1 Anti-EGFR Chemotherapy Radiotherapy	Pembrolizumab + Cisplatin/Cetuximab + Intensity modulated radiotherapy (IMRT)	2	50	2021/9/30
NCT02764593	Anti-PD-1 Anti-EGFR Chemotherapy Radiotherapy	A: Nivolumab + Cisplatin + IMRT B: Nivolumab + High Dose Cisplatin + IMRT C: Nivolumab + Cetuximab + IMRT D: Nivolumab + IMRT	1	120	2019/3/1
NCT03051906	Anti-PD-L1 Anti-EGFR Radiotherapy	Durvalumab + Cetuximab + IMRT	1,2	69	2022/1/1
NCT03292250	Anti-PD-L1 Anti-CTLA-4	Durvalumab + Tremelimumab	2	259	2020/1/1
NCT02834013	Anti-PD-1 Anti-CTLA-4	Nivolumab + Ipilimumab	2	707	2020/8/31
NCT03463161	Anti-PD-1 IDO Inhibitor	Pembrolizumab + Epacadostat	2	30	2020/3/1
NCT03325465	Anti-PD-1 IDO Inhibitor	A: Pembrolizumab B: Pembrolizumab + Epacadostat	2	44	2020/6/1
NCT03358472	Anti-PD-1 IDO Inhibitor	A: Pembrolizumab B: Pembrolizumab + Epacadostat C: Cetuximab + Cisplatin or Carboplatin + 5-fluorouracil	3	625	2021/1/27
NCT03343613	Anti-PD-L1 IDO Inhibitor	LY3381916 (IDO1 Inhibitor) + LY3300054 (Anti-PD-L1)	1	290	2019/9/1
NCT02903914	Anti-PD-1 Arginase Inhibitor	A: INCB001158 (Arginase Inhibitor) B: Pembrolizumab + INCB001158	1,2	346	2019/5/1
NCT03454451	Anti-PD-1 Anti-CD73	A: CPI-006 (CD73 Inhibitor) B: CPI-006 + CPI-444 (Adenosine-A2A Receptor Inhibitor) C: Pembrolizumab + CPI-006	1	378	2022/3/1
NCT03162224	Anti-PD-L1 Vaccine	Durvalumab + MEDI0457 (a HPV DNA vaccine)	1b/2a	40	2020/3/2
NCT02432963	Anti-PD-1 Vaccine	Pembrolizumab + p53MVA Vaccine (modified vaccinia virus Ankara vaccine expressing p53)	1	19	2018/4/1
NCT03260023	Anti-PD-L1 Vaccine	Avelumab + TG4001 (a HPV vaccine)	1,2	52	2020/5/1
NCT02526017	Anti-PD-1 Anti-CSF-1R	A: Cabiralizumab B: Nivolumab + Cabiralizumab	1	295	2019/5/1
NCT02452424	Anti-PD-1 Anti-CSF1R	Pembrolizumab + PLX3397	1,2	80	2018/5/1
NCT02335918	Anti-PD-1 CD-27 Agnoist	Nivolumab + Varlilumab	1,2	175	2019/4/1
NCT02475213	Anti-PD-1 Anti-B7-H3	Pembrolizumab + Enoblituzumab	2	75	2018/8/1
NCT02952989	Anti-PD-1 Fucosylation Inbihitor	Pembrolizumab + SGN-2FF (Fucosylation Inbihitor)	1	308	2019/12/1
NCT03474497	Anti-PD-1 IL-2 Radiotherapy	Pembrolizumab + IL-2 + Radiotherapy	1,2	45	2020/7/1
NCT03518606	Ant-PD-L1 Anti-CTLA-4 Chemotherapy	Durvalumab + Tremelimumab + Metronomic Vinorelbine	1,2	150	2020/12/29
NCT02551159	Anti-PD-L1 Anti-CTLA-4 Anti-EGFR Chemotherapy	A: Durvalumab B: Durvalumab + Tremelimumab C: Cetuximab + 5-fluorouracil + Cisplatin + Carboplatin	3	823	2018/12/31
NCT02643303	Anti-PD-L1 Anti-CTLA-4 TLR3 agonist	Durvalumab + Tremelimumab + Poly ICLC(a TLR3 agonist)	1,2	102	2022/8/1
NCT03019003	Anti PD-L1 Anti-CTLA-4 Chemotherapy	Durvalumab + Tremelimumab + Azacitidine	1B/2	59	2020/7/1
NCT03283605	Anti-PD-L1 Anti-CTLA-4 Radiotherapy	Durvalumab + Tremelimumab + Stereotactic Body Radiotherapy (SBRT)	1,2	45	2019/12/1
NCT03085914	Anti-PD-1 IDO-inhibitor Chemotherapy	A: Pembrolizumab + Epacadostat + mFOLFOX6 (oxaliplatin, leucovorin, 5-fluorouracil) B: Pembrolizumab + Epacadostat + Gemcitabine + Nab-paclitaxel C: Pembrolizumab + Epacadostat + Carboplatin + Paclitaxel D: Pembrolizumab + Epacadostat + Pemetrexed + Platinum E: Pembrolizumab + Epacadostat + Cyclophosphamide F: Pembrolizumab + Epacadostat + Gemcitabine + Platinum G: Pembrolizumab + Epacadostat + 5-fluorouracil + Platinum	1,2	421	2021/4/1
NCT03236935	Anti-PD-1 NO Synthase Inhibitor	Pembrolizumab + L-NMMA	1	12	2019/2/1
NCT03245489	Anti-PD-1 Anti-platelet	A: Pembrolizumab + Clopidogrel + Acetylsalicylic Acid Follwed by Pembrolizumab alone B: Pembrolizumab alone Follwed by Pembrolizumab + Clopidogrel + Acetylsalicylic Acid	1	20	2020/12/30
NCT02636036	Anti-PD-1 Oncolytic Virus	Nivolumab + Enadenotucirev	1	30	2019/3/1

## Combined with chemotherapy

**Figure 2:**
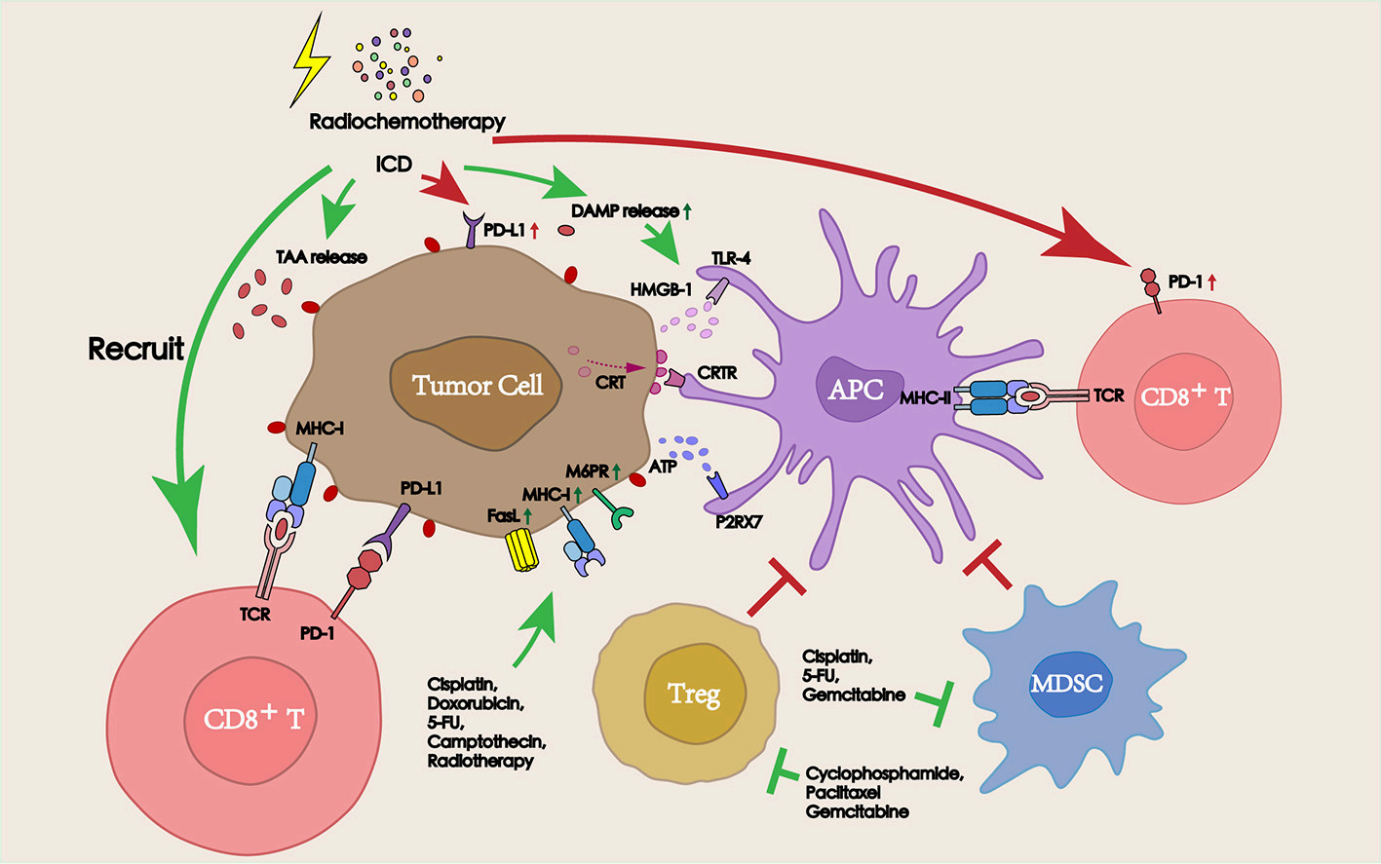
Interactions between radiochemotherapy and PD-1/PD-L1 blockade in the TME. Radiochemotherapy can increase the release of TAAs and DAMPs, improve the expression of PD-L1 on the tumor surface. Some chemotherapeutic agents can also deplete immunosuppressive cells (Tregs and MDSCs), promote the function of APCs, and increase the sensitivity of tumors to cytotoxic immune cells, thereby improving the therapeutic efficacy of PD-1/PD-L1 blockade immunotherapy.

Chemotherapy, which has been known to induce systemic immunosuppression due to the bone marrow toxicity, is used as a traditional therapy to control the growth of tumors and block the proliferation of tumor cells due to its cytotoxicity (19). However, recent studies have found that many chemotherapeutic agents exerted a stimulating effect on the antitumor immune response beyond their cytotoxicity, thereby aiding the immune system in the elimination of tumor cells (20). From the tumor side, chemotherapy can promote the release of a series of TAAs by dying cells and increase the sensitivity of tumor cells to cytotoxic immune cells to promote inflammatory response. Also, from the immune side, multiple types of chemotherapeutic agents have been shown to promote lymphocyte infiltration into the tumor site, deplete immunosuppressive cells, and directly increase the activity of antigen presenting cells (APCs). The improvement in the TME following treatment with chemotherapeutic agents suggests an attractive synergy between cytotoxic chemotherapy and PD-1/PD-L1 blockade (Figure 2).

### Improvement of tumor immunogenicity

Some antineoplastic drugs including 5-fluorouracil (5-FU) and cisplatin could increase the expression of MHC I on the surface of tumor cells (21). Moreover, the expression of tumor antigens (carcinoembryonic antigen, CEA) could also be promoted by 5-FU (21, 22). Treatment with platinum and paclitaxel could increase the production of macrophage chemoattractant protein-1 (MCP-1) thus recruiting infiltrating macrophages into the tumor site (23).

Using chemotherapeutic agents, some types of cell death have been demonstrated to induce an immune response against antigens released from dying cells, commonly referred to as immunogenic cell death (ICD): the dying tumor cells can release a series of damage-associated molecular patterns (DAMPs), including calrectin (CRT) (24), adenosine triphosphate (ATP) (25), and high mobility group box 1 (HMGB1) (26), which plays an important role in the immune response elicited by ICD. DAMPs that are exposed to a large number of dead tumor cell surfaces or released into the TME can be recognized by APCs surface receptors such as toll-like receptor-4 (TLR-4), calrectin receptor (CRTR), and purinergic receptor P2RX7 (20), thereby promoting the maturation and proliferation of APCs, and sequentially leading to the activation of cytotoxic lymphocytes.

### Sensitization of tumor cells

A variety of chemotherapeutic drugs can upregulate the expression of death receptors on the surface of tumor cells, thereby increasing the sensitivity of tumor cells to the attack from immune cells expressing death receptor ligands.

Cyclophosphamide chemotherapy could sensitize tumor cells to the tumor necrosis factor-related apoptosis-inducing ligand receptor (TRAILR) dependent CD8^+^ T cell-mediated immune attack, resulting in the suppression of tumor growth (27). Furthermore, treatment with 5-FU was demonstrated to upregulated FasR expression and sensitized tumor cells to low-avidity cytotoxic lymphocytes (28). In addition, paclitaxel, cisplatin, doxorubicin could upregulate the expression of mannose-6-phosphate receptor (M6PR) on the surface of tumor cells, thereby increasing the permeability of cell membrane to granzyme B, making the cytotoxicity of lymphocytes to tumor cells independent of perforin (29).

### Recruitment of effector cells

The recruitment and infiltration of effector cells to the tumor site determines the efficiency of the antitumor immune response and the prognosis of PD-1/PD-L1 blockade immunotherapy. Preclinical investigations suggested that chemotherapy acts as a promoter and facilitator for the recruitment and infiltration of effector cells. The administration of low-dose cisplatin and paclitaxel could also trigger the recruitment of macrophages and tumor-specific CD8^+^ T-cells into the tumor site (30). Thus, the infiltration of CD3^+^ T cells into the tumor mass increased after preconditioning with cisplatin, markedly enhancing the efficacy of adoptively transferred cytokine-induced killer (CIK) cells (31). In addition to promoting the homing of transferred lymphocytes to secondary lymphoid organs and tumor mass, treatment with cyclophosphamide treatment also favored the homeostatic proliferation/activation of transferred B and T lymphocytes (32). Moreover, sequential chemotherapy (cisplatin and gemcitabine) in mice previously treated with adenovirus-based immunotherapy increased the number and activity of both systemic and intratumoral CD8^+^ T cells (33).

### Inhibition of immunosuppressive cells

Chemotherapeutic agents, especially when applied at low-dose, may selectively inhibit immunosuppressive cells (including Tregs and MDSCs) with little adverse effects on immune effector cells.

Intratumoral and splenic Tregs in multiple types of tumors could be depleted after treatment with cisplatin, gemcitabine treatment (34), while paclitaxel chemotherapy selectively decreased the size of the Treg population rather than that of other subsets including cytotoxic T cells by upregulating Fas and Bcl-2/Bax mediated apoptosis (35, 36). Moreover, metronomic chemotherapy with paclitaxel could deplete Tregs and simultaneously inhibit tumor angiogenesis, instead of displaying direct cytolytic effects (37). Furthermore, the application of metronomic chemotherapy involving cyclophosphamide in patients with advanced tumors not only depleted Tregs, but also restored the function of T cells and NK cells (38).

Various chemotherapeutic agents showed the selective depletion of MDSCs at non-cytotoxic doses, including 5-FU, paclitaxel, gemcitabine, doxorubicin, and platinum (22). Apart from the depletion of MDSCs, treatment with ultralow-dose paclitaxel treatment also promoted the maturation of MDSCs into DCs, which exhibit a higher expression of MHC II and CD86/CD40 co-stimulatory molecules (39). A sequential treatment with gemcitabine and cisplatin could also prevent the increase in the number of systemic immunosuppressive cells induced by adenoviral-based immunotherapy. After immunotherapy, the number of MDSCs, Tregs and B cells in spleen was significantly upregulated, while the addition of chemotherapy reduced the number of those immunosuppressive cells (33).

### Promotion of APCs functions

Several chemotherapeutic agents may promote antigen presentation by directly stimulating the maturation and activation of DCs. Treatment with low-dose cyclophosphamide could increase the potency of DCs with respect to antigen presentation and cytokine secretion (40). Cyclophosphamide at a non-myeloablative doses could also promote the compensatory myelopoiesis and alter DCs homeostasis, thus leading to a higher secretion of IL-12 and a lower secretion of IL-10, and eventually priming the T cell activation and inhibiting Tregs (41). When transfected with low dose 5-FU-treated tumor RNA, DC activation markers increased, leading to a significant increase in the IFN-γ producing T cells in tumor-bearing mice (42). Moreover, the expression of the co-stimulatory factors, such as CD80, CD86, CD40, and MHC-II, on DCs could also be stimulated by various chemotherapeutic agents including vinblastine, paclitaxel, doxorubicin, and methotrexate at nontoxic concentrations, which directly enhanced DCs maturation and their activation of T cells, eventually initiating a more effective antitumor immune response (43, 44).

### Combining chemotherapy with PD-1/PD-l1 blockade

Although various chemotherapeutic agents have been shown to promote antitumor immune responses, tumor cells tend to acquire resistance after chemotherpay, which may be caused by tumor immune escape. Some chemotherapeutic drugs can lead to a high expression of PD-1/PD-L1 through the MAPK/ERK kinase pathways in patients with HNSCC (45), thereby promoting the tumor immune escape. The high expression of PD-1/PD-L1 as a result of chemotherapy provides the possibility of combining chemotherapy with PD-1/PD-L1 blockade. A large number of preclinical and clinical studies have confirmed that combination therapy is superior to monotherapy in controlling tumor growth and improving survival time.

In a syngeneic murine model of HNSCC, the concurrent use of cisplatin and PD-1/PD-L1 blockade delayed tumor growth and enhanced survival without significantly reducing the number or function of TILs or increasing the cisplatin-induced toxicity (46). Moreover, taxane and 5-FU treatment upregulated the expression of PD-L1, thus inhibiting the function of antigen-specific T cells and promoting PD-L1-mediated T cell apoptosis in breast cancer cell (47). A significant increase of PD-L1 expression was observed after docetaxel, platinum, and fluorouracil (TPF) treatment. The mean density of tumor-infiltrating CD8^+^ T cells also significantly increased after TPF treatment (45). Also, PD-L1 expression was significantly upregulated in patients with HNSCC after carboplatin chemotherapy, suggesting that these patients may benefit from the sequential PD-1/PD-L1 immunotherapy (48). In addition to increasing PD-1/PD-L1 expression and improving the TME, some chemotherapeutic agents may also induce the production of immunosuppressive cells. Despite the immunostimulatory effect of cyclophosphamide, it could also induce an increase of MDSCs (CPM-MDSCs) (49), leading to an immunosuppressive effect. Doxorubicin combined with cyclophosphamide chemotherapy could increase the number of MDSCs in breast cancer patients (50). Combining with PD-1/PD-L1 blockade could eliminate the CPM-MDSCs-mediated immunosuppression, enhance antigen-specific immune responses, increase the intratumoral ratios of CD8^+^/Tregs and CD4^+^ Foxp3^+^/Tregs, and prolong the CPM inhibition of Tregs, thus leading to a long-lasting antitumor effect (51, 52).

In summary, the above evidence suggests a strong synergistic effect between chemotherapy and PD-1/PD-L1 blockade immunotherapy. Regrettably, no studies have been conducted to compare the effects of different combinations of chemotherapy drugs with PD-1/PD-L1 blockade so far. However, low-dose, metronomic chemotherapy is recommended in this review because tumor-associated antigen presentation is promoted while the patient's immune system function can be better protected. Similarly, some chemotherapeutic drugs, such as gemcitabine, paclitaxel, have been shown to promote immune responses at tumor sites in a variety of ways in the treatment of other solid tumors, thereby synergistically interacting with PD-1/PD-L1 blockade. As common chemotherapeutic drugs for HNSCC, they are also supposed to exhibit a similar synergistic effect with PD-1/PD-L1 blockade in HNSCC, but further clinical analysis still needs to be carried out. Therefore, combining PD-1/PD-L1 with chemotherapy can be an effective strategy to improve clinical outcomes.

## Combined with radiotherapy

Radiotherapy, alone or in combination with chemotherapy plays an important role in the treatment of early and advanced HNSCC by locally controlling of tumor progression. While destroying tumor cells, radiotherapy can also damage immune cells in the affected area. Similar to chemotherapy, radiotherapy has been generally considered to inhibit the immune system in the past. Recent studies have found that radiotherapy could also induce antitumor immune responses similar to chemotherapy. While destroying tumor cells and tumor stromal cells, radiotherapy could promote the release of a large number of TAAs (53). At the same time, radiotherapy could also induce ICD, thereby promoting the activation and maturation of DCs, and upregulating MHC I expression on tumor cells (54, 55). Thus, T-cell priming in draining lymphoid tissues dramatically increased after radiotherapy, leading to the reduction of the primary tumor or distant metastases in a CD8^+^ T cell-dependent fashion (56). In addition, the increase in TILs after radiotherapy also demonstrated the activation of the immune system following radiotherapy. Therefore, considering the immunostimulatory properties of radiotherapy, a combined application of radiotherapy and immunotherapy is feasible (57).

In patients with HNSCC, fractionated conformal radiation increased the number of circulating CD8^+^ T cells, and significantly decreased serum CXCL10 and CXLC16 levels. Moreover, radiation-induced tumor lysis led to a decrease in tumor CXCL10 secretion, while the levels of CXCL16 secreted by the APCs into the circulation increased, thereby attracting T cells to the TME and increasing the number of TILs (58). However, radiotherapy also increased the number of MDSCs, Tregs, and T cells expressing checkpoint receptors (particularly PD-1) (58, 59). Interferon-γ (IFN-γ) produced by CD8^+^ T cells after ionizing radiation was responsible for mediating the upregulation of PD-L1, resulting in a PD-1/PD-L1-mediated CD8^+^ T cell inactivation and depletion (59, 60). Furthermore, the increased secretion of transforming growth factor-β (TGF-β) after radiotherapy contributed to the infiltration of Tregs into the TME and then suppressed the immune response (61, 62). Stereotactic radiotherapy in combination with PD-1 blockade could reduce the number of Tregs and increase the proportion of CD8^+^ T cells, thereby enhancing the cytotoxicity toward tumor cells (63). Combinatorial therapy could also enhance the specific antitumor immune response by cross-presenting TAAs (63), inhibit the local accumulation of MDSCs, and reconstruct the TME (64). In a murine orthotopic model of HNSCC, fractionated conformal radiotherapy could sensitize anti-PD-L1-resistant tumors, and a combination of radiotherapy with PD-L1 blockade resulted in a significant enhancement in tumor control and improvement in survival when compared with monotherapy (65).

PD-1/PD-L1 blockade in combination with radiotherapy could also suppress the metastatic tumors via abscopal effect. A combination of ablative radiotherapy with PD-1 blockade led to a 66% reduction in the size of non-irradiated tumors in mice with melanoma (66). In mice with breast cancer undergoing ionizing irradiation together with PD-L1 blockade, not only the tumor at the radiotherapy site but also the metastatic tumors outside the irradiated site were significantly reduced, and a long-lasting immune memory was elicited (67). These synergistic effects could be attributed to the fact that radiotherapy promoted the release of a large number of TAAs, thereby activating the immune system and inhibiting tumors outside of radiotherapy site.

## Combined with targeted therapy

**Figure 3:**
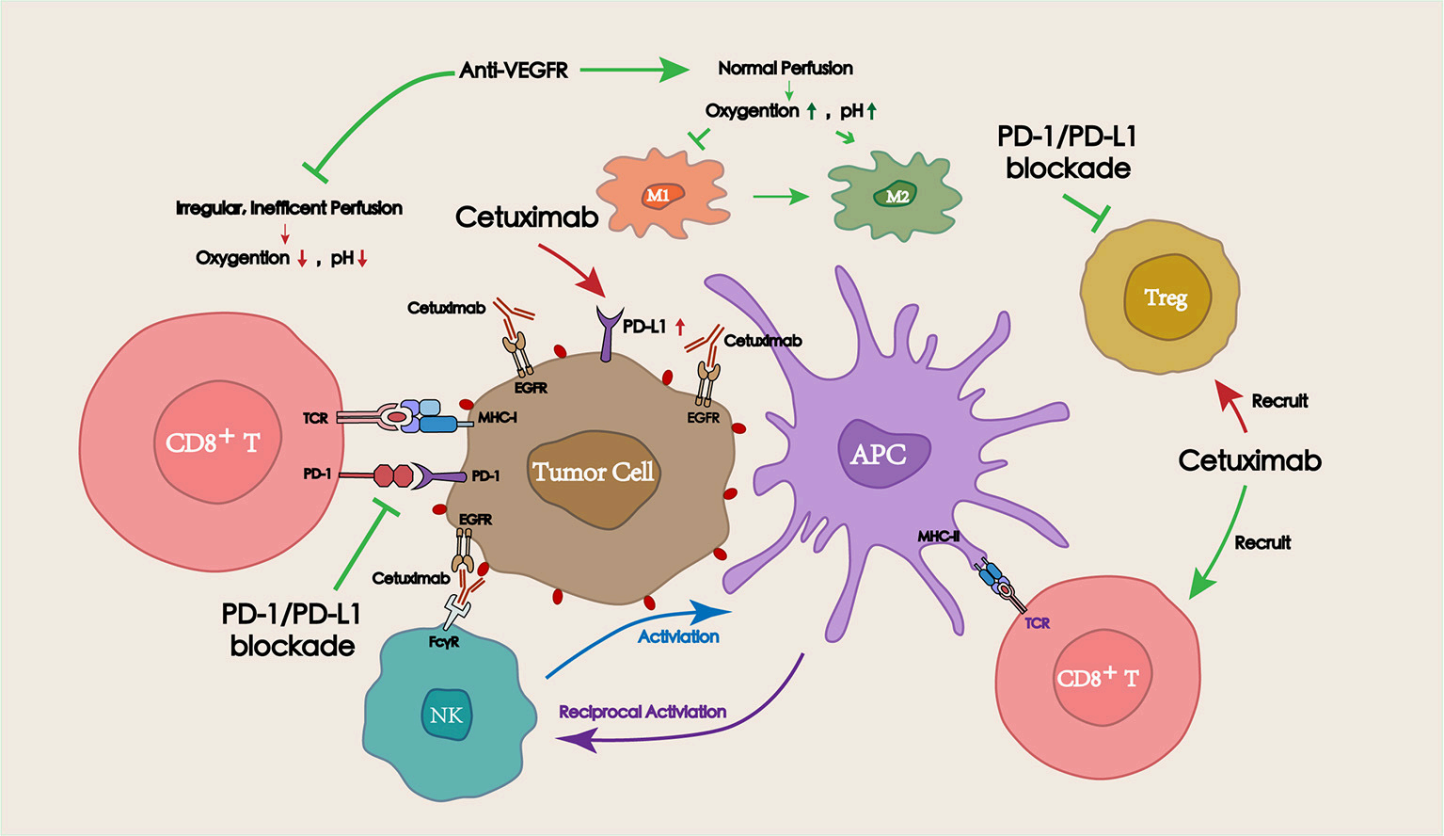
Interactions between targeted therapies (including tumor targeting IgG mAbs and vascular targeting drugs) and PD-1/PD-L1 blockade in the TME. Tumor targeting IgG mAbs (represented by cetuximab) can recruit and activate NK cells via ADCC effect, thereby lysing tumor cells. Activated NK cells can also promote antitumor immune responses by secreting cytokines to facilitate the crosstalk with dendritic cells (DCs) and other immune cells (macrophages, other NK cells). Appropriate doses of vascular targeting drugs can restore blood perfusion at the tumor site, and then improve the hypoxic and acidic TME, which is beneficial for the function of antitumor immune cells.

Several commonly used IgG1 antibodies (represented by cetuximab) have been shown to modulate the TME via antibody-dependent cellular cytotoxicity (ADCC) and promote the antitumor immune response (68, 69). Distinct from the tumor targeted IgG1 antibodies, anti-angiogenic drugs could regulate the blood perfusion into the tumor site, adjusting the oxygen concentration and the pH, and eventually affecting the TME. Combining targeted drugs with immune checkpoint inhibitors resulted in a satisfactory antitumor efficacy. Herein, the mechanisms of tumor/vascular targeting drugs in combination with PD-1/PD-L1 blockade for the treatment of HNSCC are described below (Figure 3).

### Tumor targeting mAbS

EGFR was highly expressed in various types of tumor tissues, and the overexpression of EGFR could be detected in more than 90% of HNSCC cases (70). Cetuximab, a humanized IgG1 mAb targeting EGFR, was approved by the FDA in 2006 for the treatment of head and neck malignancies. The crosstalk of ADCC effect and host immune response suggested the feasibility of combining cetuximab with immune checkpoint inhibition therapy (69).

NK cells and DCs represent the main immune cells that mediate the ADCC effect. The antibody IgG Fc receptor (FcγRIIIa) is mainly expressed on the NK cells, therefore, when cetuximab specifically binds to the antigen on the target cell through its Fab segment, the naked Fc segment binds to Fc receptors on NK cells. The antibody could act as a bridge between target cells and NK cells, activating NK cells and promoting the release of cytotoxic mediators (perforin and granzymes), eventually leading to the lysis of target cells (71). Except for NK cells, the IgG Fc receptor (FcγRIIa) has been found to be expressed on DCs and neutrophils, which may also mediate several important downstream immunological responses (72). TAAs released by the lysed tumor cells can be cross-presented by DCs to cytotoxic T cells, thereby triggering the activity of cytotoxic T cells (73). In addition, activated NK cells can secrete cytokines to promote DCs maturation. In turn, the stimulation of NK cells can be greatly enhanced through the stimulation by cytokines and surface molecules from mature DCs (72). Moreover, the crosstalk between NK cells and other immune cells is important for promoting the infiltration of cytotoxic T cells into the tumor site, which perform their lytic activity on tumor cells and further stimulate long-term immune responses (74).

Similar to chemotherapy and radiotherapy, PD-1 expression on CD8^+^ TILs significantly increased in patients treated with cetuximab; this was inversely correlated with the clinical outcome of cetuximab therapy (75). The recruitment of immunesuppressive cells after cetuximab treatment might also explain the resistance to EGFR-targeted therapy. Among patients who exhibit poor responses to cetuximab treatment, a significant increase in the number of Tregs in peripheral blood and TILs was observed (76). Moreover, there was a negative correlation between the activation of NK cells and the expansion of the Treg population, which in turn inhibited the ADCC effect (77). In a patient with poorly differentiated HNSCC, a clinical remission of invasion was observed after a combined application of cetuximab and nivolumab for 6 months (78). These evidences suggested that the anti-EGFR-resistant patients might benefit from PD-1 blockade immunotherapy. Notably, PD-L1 expression was significantly lower in EGFR-mutant tumors than that in EGFR-wild-type tumors; therefore, patients with EGFR mutations might not benefit from the combination therapy. Therefore, a combined PD-1 therapy is not recommended as a regimen to enhance the treatment efficacy in these patients (79, 80).

Consequently, treatment with cetuximab could drive ADCC and other immune effects, thus leading to immunosuppression by initiating immune checkpoints (PD-1/PD-L1 axis). When combined with PD-1 blockade, cetuximab treatment resulted in the recruitment of immune cells to the TME, while PD-1/PD-L1 blockers could attenuate the Tregs or MDSCs-mediated inhibition of effector T cells and NK cells. However, in patients with EGFR mutations, the expression of PD-L1 is downregulated, and a combined PD-L1 blockade therapy might not achieve satisfactory therapeutic effects. Therefore, the combinatorial therapy is considered to be an ideal therapeutic strategy for the treatment of EGFR-wild-type HNSSC.

### Vascular targeted drugs

The formation of tumor vessels is crucial for the process of tumor growth and metastasis, which is regulated by various cytokines, such as vascular endothelial growth factor (VEGF), angiostatin, and endostatin. Moreover, it is associated with the regulation of complex factors such as the hypoxic microenvironment, tumor suppressor genes, and oncogenes. VEGFR is highly expressed in HNSCC and associated with tumor progression and metastasis. As an independent prognostic factor, VEGFR overexpression is negatively correlated with the survival rates. Therefore, VEGFR has attracted increasing attention as an important target in HNSCC (81).

Although vascular targeting drugs treatment can prevent the development of tumors, the hypoxic environment, and acidosis environment caused by the treatment may not only affect the tumor cells, but also seriously compromise normal immune clearance function of the immune effector cells. This leads to the preferential recruitment of immunosuppressive cells that promote tumor growth, such as Tregs, MDSCs, and M2-type TAMs (81). Clinical evidence suggested that vascular targeting therapy increased PD-L1 expression and led to tumor resistance to anti-angiogenic therapy (82). The dose of vascular antagonists is closely related to its influence on the TME. The targeting of the tumor vasculature at lower vascular-normalizing doses but not high anti-angiogenic doses by an anti-VEGFR2 antibody resulted in the polarization of TAMs from an immune inhibitory M2-like phenotype toward an immune stimulatory M1-like phenotype. Moreover, it facilitated the infiltration of CD4^+^ and CD8^+^ T cells into the tumor site, thus synergizing with cancer vaccine immunotherapy (83). Normalized tumor vessels facilitated the infiltration of effector T cells, while reducing MDSC accumulation. In addition, improvements in blood perfusion polarized TAMs to an immune stimulatory M1-like phenotype, consequently re-engineering the TME and improving the efficacy of cancer immunotherapy (84). Standard (high doses) of sorafenib treatment modestly delayed the growth of tumors, but also induced hypoxia, thus increasing the tumor infiltration of Tregs, MDSCs, and M2-type TAMs, as well as the expression PD-L1. When combined with PD-L1 blockade, the activation and tumor infiltration of cytotoxic CD8^+^ T cells were enhanced (85). Furthermore, a clinical study in patients with metastatic renal cell carcinoma revealed that atezolizumab (an anti-PD-L1 antibody) combined with bevacizumab increased the number of intratumoral CD8^+^ T cells and the migration of antigen-specific T cells (86).

In summary, vascular targeting therapy can improve the TME and might enhance the therapeutic efficacy of immunotherapy, providing a new strategy for the design of PD-1/PD-L1 blockade combination therapies.

## Combined with immunotherapy

**Figure 4:**
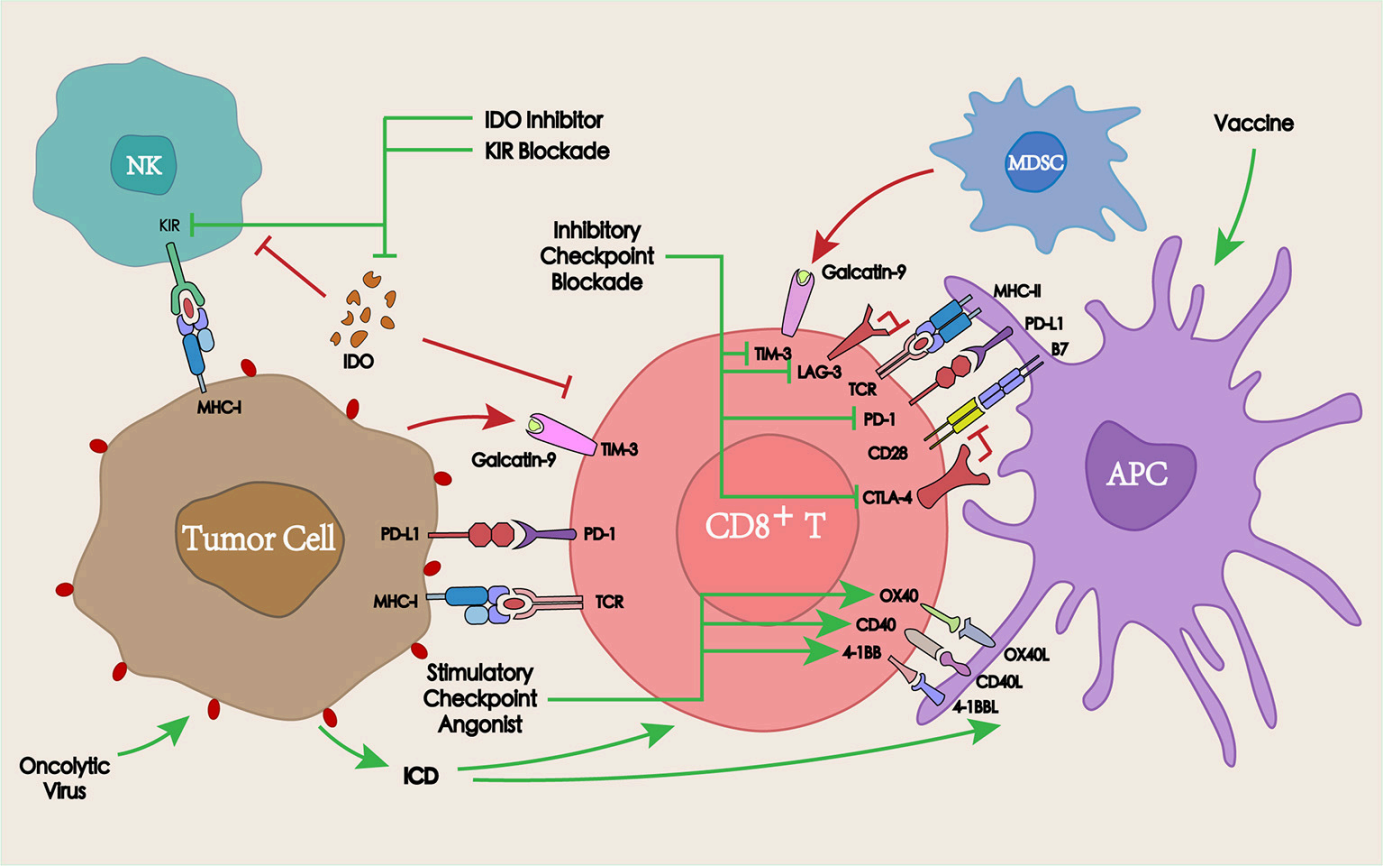
Interaction between immunotherapy and PD-1/PD-L1 blockade in the TME. PD-1/PD-L1 blockade in combination with other co-inhibitory checkpoints, co-stimulatory checkpoints, or other immunotherapies such as tumor vaccines or oncolytic viruses may overcome the tumor resistance to PD-1/PD-L1, thereby enhancing the antitumor immune response.

Pembrolizumab and nivolumab as the only immunotherapeutic drugs approved by the FDA for the treatment of platinum-refractory recurrent/metastatic HNSCC, have shown promising prospects for the treatment of advanced HNSCC. However, the immune escape mechanisms of tumors are quite complex, which always involves in multiple factors in the TME. Therefore, for different patients, personalized combined immunotherapy regimens according to pathological reports are supposed to be designed to achieve higher response rates and therapeutic effects. At present, a series of therapeutic schedules involving the combination of the PD-1/PD-L1 blockade with other co-inhibitory/co-stimulatory checkpoints or other immunotherapies are being explored (Figure 4).

### Co-inhibitory checkpoints

The activation of co-inhibitory immune checkpoints can produce a series of inhibitory signals, resulting in the inhibition of the corresponding immune response. To improve the antitumor efficacy, mutual combinations of immune checkpoint inhibitory antibodies are commonly adopted. Among these, the most widely studied is the synergistic interaction between cytotoxic T lymphocyte-associated antigen-4 (CTLA-4) blockade and PD-1 blockade.

## CTLA-4

CTLA-4 (CD152) is similar to PD-1 as an immune checkpoint molecule, which is expressed on the surface of activated CD4^+^ and CD8^+^ T cells and exerts a negative regulatory role in the initial stage of T cell activation. Since CTLA-4 ligands (CD80 and CD86) are only expressed on APCs, but not on the tumor cell surface, the CTLA-4-mediated inhibition of T cell activation occurs in secondary immune organs (lymph nodes). Unlike CTLA-4, PD-1 mainly acts on the immune microenvironment, thus a combined blockade of CTLA-4 and PD-1 may produce a synergistic effect (87).

The analysis of peripheral blood and tumor tissue specimens in patients receiving anti-PD-1, anti-CTLA-4, or the combinatorial therapy demonstrated that CTLA-4 blockade induced a proliferative signature in a subset of memory T cells. Conversely, PD-1 blockade resulted in changes in the expression of genes involved in T cells or NK cells functions (88). In a phase 1 study in patients with advanced melanoma, the ORR was 61% in the group receiving both ipilimumab and nivolumab vs. 11% in the group receiving ipilimumab and placebo, demonstrating that a combination of the two could significantly improve the ORR (89). In the treatment of refractory HNSCC with ipilimumab and nivolumab, complete remission was achieved after 4 months of therapy (90). A series of clinical trials combining CTLA-4 and PD-1/PD-L1 blockades for the treatment of HNSCC confirmed the superiority of the combinatorial therapy vs. monotherapy (Table 1).

## TIM-3

Similar to CTLA-4 and PD-1, T cell immunoglobulin-3 (TIM-3) is also one of the most studied inhibitory checkpoints. TIM-3 blockade could effectively induce antitumor immune responses by enhancing T cell effects and depleting MDSCs in a murine HNSCC model (91).

In an anti-PD-1 resistant murine tumor model, the high expression of TIM-3 on T cells was detected in TILs. Moreover, TIM-3 positive expression was also significantly correlated with anti-PD-1 treatment time. The expression of TIM-3 was low before and during the treatment-sensitive period, while it significantly increased after drug resistance (92). Treatment with anti-TIM-3 antibodies after PD-1 blockade overcame anti-PD-1 resistance and significantly increased median survival time in tumor-bearing mice. In the further clinical study, TIM-3 was also found to be highly expressed on the surface of TILs in patients with resistance to PD-1 blockade therapy, suggesting that a combined anti-TIM-3 therapy had the potential to overcome resistance to anti-PD-1 therapy (92, 93). In patients with advanced HNSCC, PD-1 blockade might promote the expression of TIM-3, which triggered the resistance to anti-PD-1 therapy in the TME. To further explore the feasibility of TIM-3 blockade for overcoming PD-1 resistance, PD-1 blockade combined with TIM-3 blockade was applied in murine HNSCC models. This combinatorial therapy significantly improved the antitumor effect when compared with anti-PD-1 monotherapy (18).

### Other co-inhibitory checkpoints

The blockade of other inhibitory immune checkpoints such as lymphocyte activation gene-3 (LAG-3), T cell immunoglobulin and ITIM domain protein (TIGIT), and their combination with PD-1/PD-L1 blockade for the treatment of malignancies are also being explored (94, 95). However, only a few studies have focused on HNSCC, and more basic experiments and clinical studies still need to be carried out.

Overall, in the treatment of HNSCC, PD-1/PD-L1 blockade may result in a further upregulation of the inhibitory checkpoints on the surface of TILs, thus supporting a circuit of compensatory signaling and potentially permitting immune escape from PD-1/PD-L1 blockade in the TME. Therefore, combining inhibitory checkpoint blockade with PD-1/PD-L1 blockade may provide an effective strategy to overcome acquired resistance to PD-1/PD-L1 blockade, as well as to increase the ORR and therapeutic efficacy.

### Co-stimulatory checkpoints

Unlike co-inhibitory checkpoints, co-stimulatory checkpoints can induce immunostimulatory effects by activating members of the tumor necrosis factor receptor superfamily on T cells (96). The most studied stimulatory checkpoint receptors and their ligands to date are mainly OX40, 4-1BB, and CD27, whose agonist in combination with PD-1/PD-L1 blockade are also being developed for the treatment of HNSCC (97, 98).

## OX40

OX40 and its ligand OX40L are important co-stimulatory molecules in the immune response, which can improve the immunosuppressive effect in the TME and enhance the cytotoxicity of the effector lymphocytes (99). In a PD-1 treatment-resistant murine model of ovarian cancer, PD-1 blockade combined with an OX40 agonistic antibody treatment led to a promising ORR of 60% (100).

## CD27

Similar to OX40L, CD70 (ligand of CD27) is found on activated T cells, leading to CD27-CD70 interactions that may involve direct cellular communication between T cells and APCs (101). CD27 agonists synergized with PD-L1 blockade by enhancing CD8^+^ T cell proliferation and effector cytokine generation, while simultaneously downregulating the expression of multiple quiescence-related genes (102). In human CD27 transgenic mice, varlilumab (an CD27 agonist mAb), similarly synergized with PD-L1 blockade in protecting against lymphoma (103). An ongoing phase I/II clinical trial (NCT02335918) is designed to determine the clinical benefit, safety, and tolerability of combining varlilumab and nivolumab in advanced refractory solid tumors including HNSCC.

## 4-1BB

4-1BB (CD137) is expressed on a variety of immune cells including T cells, B cells, NK cells, and DCs. 4-1BB signaling can enhance the proliferation and activation of T ligands bitory PD-1 and TIM-3 checkpoints (104). For advanced giant tumors that could not be controlled by anti-PD-1 monotherapy, its combination with CD137 agonist therapy resulted in a complete rejection (105). Moreover, a phase Ib study (NCT02179918) has already demonstrated the safety, tolerability, and clinical activity of utomilumab (a 4-1BB agonist mAb) in combination with pembrolizumab in the treatment of advanced solid tumors including HNSCC (106).

### Other immunotherapies

In addition to immune checkpoints, other therapies targeting the immune system, such as the indoleamine 2,3-dioxygenase 1 (IDO1) inhibitor, tumor vaccines, oncolytic viruses, and NK cell Ig-like receptor (KIR) in combination with PD-1/PD-L1 blockade are also under development.

IDO1 is a rate-limiting enzyme in the catabolism of tryptophan, which can induce the apoptosis or dysfunction of T cells and NK cells, thereby weakening the antitumor immune response. The inhibition of IDO1 activity could restore the function of T cells and NK cells and overcome the tumor immune tolerance (107). In patients with oral squamous cell carcinoma, PD-L1 and IDO1 were overexpressed. Moreover, the expression of PD-L1 and IDO1 was higher in patients without alcohol and tobacco history, suggesting that patients with HNSCC that do not smoke or drink may benefit more from PD-L1 and IDO1 inhibition (108). In a phase II clinical trial, a limited benefit from the PD-1 blockade was observed in selected patients with advanced soft-tissue sarcoma and gastrointestinal stromal tumors. This could be explained by an immunosuppressive TME resulting from macrophage infiltration and the activation of IDO1 pathway (109). A combined PD-L1 and IDO1 blockade could increase the percentage of CD8^+^ T cells in TILs and enhance the antitumor effects and survival time (110). A series of clinical trials combining IDO inhibitors with PD-1/PD-L1 blockade in the treatment of head and neck cancers are currently underway (NCT03463161, NCT03325465, NCT03358472, NCT03343613).

Cancer vaccines could provide antigens to activate the immune system, initiate antitumor immune responses, and overcome tumor-induced immunosuppression (111). Although tumor vaccine-induced immune responses have been observed in most studies, in clinical studies tumor vaccines were insufficient as a monotherapy. Therefore, combining tumor vaccines with other therapies such as PD-1/PD-L1 blockade might lead to better clinical outcomes (112), and several ongoing clinical trials are investigating the efficacy of tumor vaccines in combination with PD-1/PD-L1 blockade for the treatment of HNSCC (NCT03162224, NCT02432963, NCT03260023).

Oncolytic virus therapy represents a novel tumor immunotherapy that utilizes the virus to specifically replicate in tumor cells resulting in the lysis of tumor cells, which can lead to ICD and stimulate specific antitumor immune responses (113). Talimogene laherparepvec (TVEC) is a genetically modified type I herpes simplex virus engineered to selectively replicate within tumors and to produce the granulocyte-macrophage colony stimulation factor (GM-CSF), thus enhancing systemic antitumor immune responses (114). The results of a phase I clinical trial showed that combining an oncolytic virus with pembrolizumab could improve the efficacy of anti-PD-1 therapy by improving the TME, without increasing the toxicity in patients with advanced melanoma (115). An ongoing clinical trial has been established to further evaluate the safety and efficacy of the combination of TVEC with pembrolizumab for the treatment of R/M HNSCC (NCT02626000).

## Specific landscape for HNSCC

In addition to the above-mentioned several commonly applied solid tumor treatment strategies, due to the specific etiology of HNSCC, some treatments for HNSCC have also been developed, and these therapies are blocked with PD-1/PD-L1. The efficacy of the combined application is also being evaluated.

Tobacco, alcohol consumption, and human papillomavirus infection (HPV) are considered to be the major risk factors for HNSCC. Toxic-induced and HPV-induced HNSCC are described as two different clinical entities with different oncologic pathways and prognosis in the 2017 WHO classification (116). Furthermore, there is also a high rate of somatic mutation in HNSCC (117). Therefore, the immunogenicity of HNSCC differs depending on tumor intrinsic antigen, neoantigen derived from the mutations, or the expression of the antigen induced by the HPV infection. Tumor immune microenvironment also differs in terms of the mutations load and viral infection (118). Cancer genomic analysis of different patients is important for the design of PD-1/PD-L1 blockade combinatorial therapy.

### HPV infection in combinatorial therapy in PD-1/PD-l1 blockade

As HPV-induced HNSCC is considered as a distinct clinical entity in terms of clinical presentation, response to treatment and prognosis, the specificities in its microenvironment need to be more precisely characterized in order to stratify patients. Due to the specificity of HPV-induced HNSCC in clinical manifestations and response to treatment, there are significant differences in tumor immune microenvironment among HNSCC patients based on HPV infection status, thus the ORRs to PD-1/PD-L1 blockade are also different.

HPV-positive HNSCC samples showed a significantly higher number of invasive CTLs, dendritic cells and pro-inflammatory chemokines, which was considered to be positively correlated with a favorable prognosis. In addition, TIL in HPV-positive tumors has significantly higher PD-1 expression (119, 120). In patients treated with pembrolizumab for advanced HNSCC, the response rate for HPV-positive populations was 32%, compared with 14% for HPV-negative populations. Of patients that responded, the 6-months progression-free survival rate for HPV-positive population was 37%, compared with 20% for HPV-negative populations, suggesting that the efficacy of PD-1/PD-L1 blockade is related to HPV infection status (14). In the treatment of E6/E7 expressing ovarian cancer burden mice, when HPV-E6/E7 vaccine was combined with PD-L1 blockade, most tumor growth was effectively controlled (121). However, the ORR was only 33% when treating HNSCC patients with HPV vaccine and PD-1 blockade (122). To the inconsistency with the results of preclinical studies, the investigators speculated that although the HPV-16-specific T cells increased after vaccine injection, these vaccine-induced T cell populations may be necessary, but not sufficient to enhance the ORR in combination of nivolumab, and additional immunosuppressive pathways might still exist. Further clinical trials to verify the superiority of the HPV vaccine in combination with PD-1 blockade are still needed.

### Cancer genomics for designing combinatorial strategies

High-load mutations in HNSCC can lead to the expression of neoantigens and changes in TME, which may serve as new therapeutic targets. And the detection of common tumor mutation genes can also provide a reliable basis for the choice of treatment plan for patients with HNSCC. Studies on cancer genomics showed the loss of cell cycle regulation was an important driver for HNSCCs.

The most reported cell cycle regulation genes and their molecular pathways include *TP53*, pRB, CCND1, and CKN2A (123, 124). HPV infection also plays an important role in HNSCC oncogensis. The E6 and E7 oncogenes in HPV can lead to mutation and inactivation of TP53 and pRB, respectively (125). In fact, mutations in TP53 are detected in most HPV-negative HNSCC cases (126). Likewise, CDKN2A is inactivated by mutation or methylation in most HPV-negative HNSCC cases. Also, 32% of HPV-negative HNSCCs had CCND1 amplification whereas only 6% of that was observed in HPV-positive samples (123). Similarly, increased cell growth and proliferation pathway activation also plays an important role in HNSCC tumorigenesis. One of the key regulators of proliferation identified in HNSCC is EGFR. Other common mutant genes in cell growth pathway, including PIK3CA, PTEN, FGFR, and other growth factor receptors or their ligands, are also important for tumorigenesis (123).

PD-L1 membranous expression on tumor cells can result either from an adaptive immune phenomenon or from intrinsic oncogenic events in HNSCC. Several oncogenic mechanisms have been associated with PD-L1 expression, such as tumor-suppressive PTEN gene mutation or deletion, pro-oncogenic PI3K path activation, AKT/mTOR, NF-kB, and MAPK (mitogen-activated protein kinase, MEK/ERK) pathways deregulation. Either adaptive immune responses or mutations from endogenous carcinogenesis-related genes in HNSCC can lead to the upregulation of PD-L1 expression on tumor cells. Several carcinogenesis mechanisms are associated with PD-L1 expression, including tumor suppressor PTEN gene mutations or deletions, oncogene PI3K pathway activation, AKT/mTOR, NF-kB and MAPK pathway dysregulation (127).

Based on the researches on the HNSCC cancer genomics, several targeted drugs have been developed and extended to clinical applications such as EGFR which has been described above. Gene therapy drugs such as liposome or virus based TP53 gene delivers have also been developed for treatment of HNSCC (128, 129). It has been proved that transduction of p53 gene to HNSCC cells induced loss of cell viability while increased the immunogenicity and expression of PD-L1 on tumor cells, which synergized with PD-1 blockade (128).

## Future perspectives

PD-1 blockade could prevent the negative regulatory signals generated by T cells to relieve immunosuppression and enhance the antitumor efficacy of T cells. In addition, it may also abnormally enhance the autoimmune response, thereby leading to an imbalance of immune tolerance. Common autoimmune inflammatory reactions induced by PD-1 blockade involving normal tissues includes the skin symptoms, hypothyroidism, and hyperthyroidism (130). When combined with other therapies, although the therapeutic efficacy is enhanced, more serious side effects may also be triggered, for example an increase in the incidence of fatal myocarditis (131). Targeted delivery can be achieved by loading the anti-PD-1/PD-L1 antibodies within engineered carriers, thereby reducing the side effects of the PD-1/PD-L1 blockade combinatorial therapy and improving antitumor efficacy. PD-1/PD-L1 inhibitors and other drugs could be loaded into the nanoparticles to enrich drugs at the tumor site (132), or encapsulated into the hydrogels for local treatment by peritumoral injections (133). Satisfactory therapeutic outcomes effects with reduced off-target and adverse effects have been achieved, demonstrating that smart delivery of PD-1/PD-L1 inhibitors in combination with other drugs via engineered carriers could represent a promising strategy for reducing side effects as well as enhancing antitumor efficacy.

In conclusion, PD-1/PD-L1 blockade has shown good and long-lasting therapeutic effects in the treatment of HNSCC and other malignancies, but only a small subset of patients can benefit from the monotherapy. To enhance the response rate and the therapeutic effect of PD-1/PD-L1 blockade, numerous preclinical experiments, and clinical trials exploring the combinatorial therapies involving PD-1/PD-L1 blockade are underway. In this review, we summarized the current status of the combined application of chemotherapy, radiotherapy, targeted therapy, and immunotherapy in combination with PD-1/PD-L1 blockade in the field of HNSCC. Moreover, the potential mechanisms underlying the crosstalk process between PD-1/PD-L1 blockade and combinatorial cancer therapies in the TME was described. Furthermore, it is hoped that the improved understanding of the crosstalk would provide further ideas for the design of PD-1/PD-L1 combinatorial therapies for HNSCC and other malignancies.

## Author contributions

WL wrote the manuscript. MC collected the literature and generated the figures and table. LH edited and checked the manuscript format. HZ and QC reviewed the manuscript.

### Conflict of interest statement

The authors declare that the research was conducted in the absence of any commercial or financial relationships that could be construed as a potential conflict of interest.

## References

[1] Ferlay J Soerjomataram I Dikshit R Eser S Mathers C Rebelo M. Cancer incidence and mortality worldwide: sources, methods and major patterns in GLOBOCAN 2012. Int J Cancer (2015) 136:E359–86. 10.1002/ijc.29210 25220842

[2] KPrice AR, Cohen EE, Current treatment options for metastatic head and neck cancer. Curr Treatm Opt Oncol. (2012) 13:35–46. 10.1200/JCO.2015.62.0963 22252884

[3] Adelstein D Gillison ML Pfister DG Spencer S Adkins D Brizel DM. NCCN guidelines insights: head and neck cancers, version 2.2017. J Natl Compr Canc Netw. (2017) 15:761–70. 10.6004/jnccn.2017.0101 28596256

[4] Ferris RL Blumenschein G Jr Fayette J Guigay J Colevas AD. Nivolumab for recurrent squamous-cell carcinoma of the head and neck. N Engl J Med. (2016) 375:1856–67. 10.1056/NEJMoa1602252 27718784 PMC5564292

[5] Kim R Emi M Tanabe K. Cancer immunoediting from immune surveillance to immune escape. Immunology (2007) 121:1–14. 10.1111/j.1365–2567.2007.02587.x 17386080 PMC2265921

[6] Cavalieri S Rivoltini L Bergamini C Locati LD Licitra L Bossi P. Immuno-oncology in head and neck squamous cell cancers: news from clinical trials, emerging predictive factors and unmet needs. Cancer Treatm Rev. (2018) 65:78–86. 10.1016/j.ctrv.2018.03.003 29574334

[7] Stevanovic S Pasetto A Helman SR Gartner JJ Prickett TD Howie B, Landscape of immunogenic tumor antigens in successful immunotherapy of virally induced epithelial cancer. Science (2017) 356:200–5. 10.1126/science.aak9510 28408606 PMC6295311

[8] Mandal R Senbabaoglu Y Desrichard A Havel JJ Dalin MG Riaz N. The head and neck cancer immune landscape and its immunotherapeutic implications. JCI Insight (2016) 1:e89829. 10.1172/jci.insight.89829 27777979 PMC5070962

[9] McGee HS Yagita H Shao Z Agrawal DK. Programmed Death-1 antibody blocks therapeutic effects of T-regulatory cells in cockroach antigen-induced allergic asthma. Am J Respir Cell Mol Biol. (2010) 43:432–42. 10.1165/rcmb.2009–0258OC 19901343 PMC2951873

[10] Han L Liu F Ruping LI Zhaoming LI Chen X Zhou Z. Role of programmed death ligands in effective T-cell interactions in extranodal natural killer/T-cell lymphoma. Oncol Lett. (2014) 8:1461. 10.3892/ol.2014.2356 25202350 PMC4156194

[11] Ferris RL. Immunology and immunotherapy of head and neck cancer. J Clin Oncol. (2015) 33:3293–04. 10.1200/JCO.2015.61.1509 26351330 PMC4586169

[12] Zandberg DP Strome SE. The role of the PD-L1:PD-1 pathway in squamous cell carcinoma of the head and neck. Oral Oncol. (2014) 50:627–32. 10.1016/j.oraloncology.2014.04.003 24819861

[13] Yu GT Bu LL Huang CF Zhang WF Chen WJ Gutkind JS. PD-1 blockade attenuates immunosuppressive myeloid cells due to inhibition of CD47/SIRPalpha axis in HPV negative head and neck squamous cell carcinoma. Oncotarget (2015) 6:42067–80. 10.18632/oncotarget.5955 26573233 PMC4747210

[14] Chow LQ Haddad R Gupta S Mahipal A Mehra R Tahara M. Antitumor activity of pembrolizumab in biomarker-unselected patients with recurrent and/or metastatic head and neck squamous cell carcinoma: results from the phase Ib KEYNOTE-012 expansion cohort. J Clin Oncol. (2016) 34:3838–45. 10.1200/JCO.2016.68.1478 27646946 PMC6804896

[15] Chen DS Mellman I. Elements of cancer immunity and the cancer–immune set point. Nature (2017) 541:321–30. 10.1038/nature21349 28102259

[16] Brahmer J Reckamp KL Baas P Crinò L Eberhardt EEW Poddubskaya E Antonia S. Nivolumab versus docetaxel in advanced squamous-cell non–small-cell lung cancer. N Engl J Med. (2015) 373:1627. 10.1056/NEJMoa1507643 26028407 PMC4681400

[17] Weber JS D'Angelo SP Minor D Hodi FS Gutzmer R Neyns B. Nivolumab versus chemotherapy in patients with advanced melanoma who progressed after anti-CTLA-4 treatment (CheckMate 037): a randomised, controlled, open-label, phase 3 trial. Lancet Oncol. (2015) 16:375–84. 10.1016/S1470–2045(15)70076–8 25795410

[18] Shayan G Srivastava R Li J Schmitt N Kane LP Ferris RL. Adaptive resistance to anti-PD1 therapy by Tim-3 upregulation is mediated by the PI3K-Akt pathway in head and neck cancer. Oncoimmunology (2017) 6:e1261779. 10.1080/2162402x.2016.1261779 28197389 PMC5283618

[19] Nowak AK Robinson BW Lake RA. Synergy between chemotherapy and immunotherapy in the treatment of established murine solid tumors. Cancer Res. (2003) 63:4490–6. 12907622

[20] Chen G Emens LA. Chemoimmunotherapy: reengineering tumor immunity. Cancer Immunol Immunother. (2013) 62:203–16. 10.1007/s00262–012-1388–0 23389507 PMC3608094

[21] Ohtsukasa S Okabe S Yamashita H Iwai T Sugihara K. Increased expression of CEA and MHC class I in colorectal cancer cell lines exposed to chemotherapy drugs. J Cancer Res Clin Oncol. (2003) 129:719–26. 10.1007/s00432–003-0492–0 14564514 PMC12161929

[22] Aquino A Formica V Prete SP Correale PP Massara MC Turriziani M. Drug-induced increase of carcinoembryonic antigen expression in cancer cells. Pharmacol Res. (2004) 49:383–96. 10.1016/j.phrs.2003.12.007 14998548

[23] Geller MA Bui-Nguyen TM Rogers LM Ramakrishnan S. Chemotherapy induces macrophage chemoattractant protein-1 production in ovarian cancer. Int J Gynecol Cancer (2010) 20:918–25. 10.1111/igc.0b013e3181e5c442 20683396

[24] Obeid M Tesniere A Ghiringhelli F Fimia GM Apetoh L Perfettini JL. Calreticulin exposure dictates the immunogenicity of cancer cell death. Nat Med. (2007) 13:54–61. 10.1038/nm1523 17187072

[25] Ma Y Adjemian S Mattarollo SR Yamazaki T Aymeric L Yang H. Anticancer chemotherapy-induced intratumoral recruitment and differentiation of antigen-presenting cells. Immunity (2013) 38:729–41. 10.1016/j.immuni.2013.03.003 23562161

[26] Apetoh L Ghiringhelli F Tesniere A Obeid M Ortiz C Criollo A. Toll-like receptor 4-dependent contribution of the immune system to anticancer chemotherapy and radiotherapy. Nat Med. (2007) 13:1050–9. 10.1038/nm1622 17704786

[27] van der Most RG Currie AJ Cleaver AL Salmons J Nowak AK Mahendran S. Cyclophosphamide chemotherapy sensitizes tumor cells to TRAIL-dependent CD8 T cell-mediated immune attack resulting in suppression of tumor growth. PLoS ONE (2009) 4:e6982. 10.1371/journal.pone.0006982 19746156 PMC2734989

[28] Yang S Haluska FG. Treatment of melanoma with 5-fluorouracil or dacarbazine in vitro sensitizes cells to antigen-specific CTL lysis through perforin/granzyme- and Fas-mediated pathways. J Immunol. (2004) 172:4599–608. 10.4049/jimmunol.172.7.4599 15034078

[29] Ramakrishnan R Assudani D Nagaraj S Hunter T Cho HI Antonia S. Chemotherapy enhances tumor cell susceptibility to CTL-mediated killing during cancer immunotherapy in mice. J Clin Invest. (2010) 120:1111–24. 10.1172/jci40269 20234093 PMC2846048

[30] Chang C-L, Hsu T.-Y, Wu C.-C, Lai Y.-Z, Wang C, Yang Y.-C, et al. Dose-Dense chemotherapy improves mechanisms of antitumor immune response. Cancer Res. (2013) 73:119–127. 10.1158/0008–5472.can-12–2225 23108141 PMC3537885

[31] Huang X Chen YT Song HZ Huang GC Chen LB. Cisplatin pretreatment enhances anti-tumor activity of cytokine-induced killer cells. World J Gastroenterol. (2011) 17:3002–11. 10.3748/wjg.v17.i25.3002 21799646 PMC3132251

[32] Bracci L Moschella F Sestili P La Sorsa V Valentini M Canini I. Cyclophosphamide enhances the antitumor efficacy of adoptively transferred immune cells through the induction of cytokine expression, B-Cell and T-Cell homeostatic proliferation, and specific tumor infiltration. Clin Cancer Res. (2007) 13:644–53. 10.1158/1078–0432.ccr-06–1209 17255288

[33] Fridlender ZG Sun J Singhal S Kapoor V Cheng G Suzuki E. Chemotherapy delivered after viral immunogene therapy augments antitumor efficacy via multiple immune-mediated mechanisms. Mol Ther. (2010) 18:1947–59. 10.1038/mt.2010.159 20683443 PMC2990510

[34] Bracci L Schiavoni G Sistigu A Belardelli F. Immune-based mechanisms of cytotoxic chemotherapy: implications for the design of novel and rationale-based combined treatments against cancer. Cell Death Different. (2014) 21:15–25. 10.1038/cdd.2013.67 23787994 PMC3857622

[35] Zhang L Dermawan K Jin M Liu R Zheng H Xu L. Differential impairment of regulatory T cells rather than effector T cells by paclitaxel-based chemotherapy. Clin Immunol. (2008) 129:219–29. 10.1016/j.clim.2008.07.013 18771959

[36] Liu N Zheng Y Zhu Y Xiong S Chu Y. Selective impairment of CD4+CD25+Foxp3+ regulatory T cells by paclitaxel is explained by Bcl-2/Bax mediated apoptosis. Int Immunopharmacol. (2011) 11:212–9. 10.1016/j.intimp.2010.11.021 21115120

[37] Chen CA Ho CM Chang MC Sun WZ Chen YL Chiang YC. Metronomic chemotherapy enhances antitumor effects of cancer vaccine by depleting regulatory T lymphocytes and inhibiting tumor angiogenesis. Mol Ther. (2010) 18:1233–43. 10.1038/mt.2010.34 20372107 PMC2889744

[38] Ghiringhelli F Menard C Puig PE Ladoire S Roux S Martin F. Metronomic cyclophosphamide regimen selectively depletes CD4+CD25+ regulatory T cells and restores T and NK effector functions in end stage cancer patients. Cancer Immunol Immunother. (2007) 56:641–8. 10.1007/s00262–006-0225–8 16960692 PMC11030569

[39] Michels T Shurin GV Naiditch H Sevko A Umansky V Shurin MR. Paclitaxel promotes differentiation of myeloid-derived suppressor cells into dendritic cells *in vitro* in a TLR4-independent manner. J Immunotoxicol. (2012) 9:292–300. 10.3109/1547691X.2011.642418 22283566 PMC3386478

[40] Nakahara T Uchi H Lesokhin AM Avogadri F Rizzuto GA Hirschhorn-Cymerman D. Cyclophosphamide enhances immunity by modulating the balance of dendritic cell subsets in lymphoid organs. Blood (2010) 115:4384–92. 10.1182/blood-2009-11-251231 20154220 PMC2881499

[41] Radojcic V Bezak KB Skarica M Pletneva MA Yoshimura K Schulick RD. Cyclophosphamide resets dendritic cell homeostasis and enhances antitumor immunity through effects that extend beyond regulatory T cell elimination. Cancer Immunol Immunother. (2010) 59:137–48. 10.1007/s00262–009-0734–3 19590872 PMC3103867

[42] De Almeida CV Zamame JA Romagnoli GG Rodrigues CP Magalhaes MB Amedei A. Treatment of colon cancer cells with 5-fluorouracil can improve the effectiveness of RNA-transfected antitumor dendritic cell vaccine. Oncol Rep. (2017) 38:561–8. 10.3892/or.2017.5692 28586072

[43] Kaneno R Shurin GV Tourkova IL Shurin MR. Chemomodulation of human dendritic cell function by antineoplastic agents in low noncytotoxic concentrations. J Transl Med. (2009) 7:58. 10.1186/1479–5876-7–58 19591684 PMC2716306

[44] Shurin GV Tourkova IL Kaneno R Shurin MR. Chemotherapeutic agents in noncytotoxic concentrations increase antigen presentation by dendritic cells via an IL-12-dependent mechanism. J Immunol. (2009) 183:137–44. 10.4049/jimmunol.0900734 19535620 PMC4005417

[45] Ock CY Kim S Keam B Kim S Ahn YO Chung EJ. Changes in programmed death-ligand 1 expression during cisplatin treatment in patients with head and neck squamous cell carcinoma. Oncotarget (2017) 8:97920–27. 10.18632/oncotarget.18542 29228662 PMC5716702

[46] Tran L Allen CT Xiao R Moore E Davis R Park SJ. Cisplatin alters antitumor immunity and synergizes with PD-1/PD-L1 inhibition in head and neck squamous cell carcinoma. Cancer Immunol Res. (2017) 5:1141–51. 10.1158/2326–6066.cir-17–0235 29097421 PMC5712281

[47] Zhang P Su DM Liang M Fu J. Chemopreventive agents induce programmed death-1-ligand 1 (PD-L1) surface expression in breast cancer cells and promote PD-L1-mediated T cell apoptosis. Mol Immunol. (2008) 45:1470–6. 10.1016/j.molimm.2007.08.013 17920123

[48] Leduc C Adam J Louvet E Sourisseau T Dorvault N Bernard M. TPF induction chemotherapy increases PD-L1 expression in tumour cells and immune cells in head and neck squamous cell carcinoma. ESMO Open (2018) 3:e000257. 10.1136/esmoopen-2017–000257 29344407 PMC5761289

[49] Mikyšková R Indrová M Polláková V Bieblová J Símová J Reiniš M Cyclophosphamide-induced myeloid-derived suppressor cell population is immunosuppressive but not identical to myeloid-derived suppressor cells induced by growing TC-1 tumors. J Immunother. (2012) 35:374–84. 10.1097/CJI.0b013e318255585a 22576342

[50] Diaz-Montero CM Salem ML Nishimura MI Garrett-Mayer E Cole DJ Montero AJ. Increased circulating myeloid-derived suppressor cells correlate with clinical cancer stage, metastatic tumor burden, and doxorubicin-cyclophosphamide chemotherapy. Cancer Immunol Immunother. (2009) 58:49–59. 10.1007/s00262–008-0523–4 18446337 PMC3401888

[51] Mkrtichyan M Najjar YG Raulfs EC Abdalla MY Samara R Rotem-Yehudar R. Anti-PD-1 synergizes with cyclophosphamide to induce potent anti-tumor vaccine effects through novel mechanisms. Eur J Immunol. (2011) 41:2977–86. 10.1002/eji.201141639 21710477 PMC7020689

[52] Ding ZC Munn DH Zhou G. Chemotherapy-induced myeloid suppressor cells and antitumor immunity: the Janus face of chemotherapy in immunomodulation. Oncoimmunology (2014) 3:e954471. 10.4161/21624011.2014.954471 25610747 PMC4292425

[53] Jiang W Chan CK Weissman IL B.Kim YS Hahn SM. Immune priming of the tumor microenvironment by radiation. Trends Cancer (2016) 2:638–45. 10.1016/j.trecan.2016.09.007 28741502

[54] Reits EA Hodge JW Herberts CA Groothuis TA Chakraborty M Wansley EK. Radiation modulates the peptide repertoire, enhances MHC class I expression, and induces successful antitumor immunotherapy. J Exp Med. (2006) 203:1259–71. 10.1084/jem.20052494 16636135 PMC3212727

[55] Gupta A Probst HC Vuong V Landshammer A Muth S Yagita H. Radiotherapy promotes tumor-specific effector CD8+ T cells via dendritic cell activation. J Immunol. (2012) 189:558. 10.4049/jimmunol.1200563 22685313

[56] Lee Y Auh SL Wang Y Burnette B Wang Y Meng Y. Therapeutic effects of ablative radiation on local tumor require CD8+ T cells: changing strategies for cancer treatment. Blood (2009) 114:589. 10.1182/blood-2009–02-206870 19349616 PMC2713472

[57] Chakraborty M Abrams SI Camphausen K Liu K Scott T Coleman CN. Irradiation of tumor cells up-regulates Fas and enhances CTL lytic activity and CTL adoptive immunotherapy. J Immunol. (2003) 170:6338–47. 10.4049/jimmunol.170.12.6338 12794167

[58] Sridharan V Margalit DN Lynch SA Severgnini M Zhou J Chau NG. Definitive chemoradiation alters the immunologic landscape and immune checkpoints in head and neck cancer. Br J Cancer (2016) 115:252–60. 10.1038/bjc.2016.166 27380136 PMC4947695

[59] Dovedi SJ Adlard AL Lipowska-Bhalla G McKenna C Jones S Cheadle EJ. Acquired resistance to fractionated radiotherapy can be overcome by concurrent PD-L1 blockade. Cancer Res. (2014) 74:5458–68. 10.1158/0008–5472.can-14–1258 25274032

[60] Oweida A Lennon S Calame D Korpela S Bhatia S Sharma J. Ionizing radiation sensitizes tumors to PD-L1 immune checkpoint blockade in orthotopic murine head and neck squamous cell carcinoma. Oncoimmunology (2017) 6:e1356153. 10.1080/2162402x.2017.1356153 29123967 PMC5665079

[61] Kachikwu EL Iwamoto KS Liao YP DeMarco JJ Agazaryan N Economou JS. Radiation enhances regulatory T cell representation. Int J Radiat Oncol Biol Phys. (2011) 81:1128–35. 10.1016/j.ijrobp.2010.09.034 21093169 PMC3117954

[62] Sakaguchi S Yamaguchi T Nomura T Ono M. Regulatory T cells and immune tolerance. Cell (2008) 133:775–87. 10.1016/j.cell.2008.05.009 18510923

[63] Sharabi AB Nirschl CJ Kochel CM Nirschl TR Francica BJ Velarde E. Stereotactic radiation therapy augments antigen-specific PD-1-mediated antitumor immune responses via Cross-presentation of tumor antigen. Cancer Immunol Res. (2015) 3:345–55. 10.1158/2326–6066.CIR-14–0196 25527358 PMC4390444

[64] Deng L Liang H Burnette B Weicheslbaum RR Fu YX. Radiation and anti-PD-L1 antibody combinatorial therapy induces T cell-mediated depletion of myeloid-derived suppressor cells and tumor regression. Oncoimmunology (2014) 3:e28499. 10.4161/onci.28499 25050217 PMC4063144

[65] Kikuchi M Clump DA Srivastava RM Sun L Zeng D Diaz-Perez JA. Preclinical immunoPET/CT imaging using Zr-89-labeled anti-PD-L1 monoclonal antibody for assessing radiation-induced PD-L1 upregulation in head and neck cancer and melanoma. Oncoimmunology (2017) 6:e1329071. 10.1080/2162402x.2017.1329071 28811971 PMC5543907

[66] Park SS Dong H Liu X Harrington SM Krco CJ Grams MP. PD-1 Restrains radiotherapy-induced abscopal effect. Cancer Immunol Res. (2015) 3:610–9. 10.1158/2326–6066.cir-14–0138 25701325 PMC4827718

[67] Deng L Liang H Burnette B Beckett M Darga T Weichselbaum RR. Irradiation and anti-PD-L1 treatment synergistically promote antitumor immunity in mice. J Clin Invest. (2014) 124:687–95. 10.1172/jci67313 24382348 PMC3904601

[68] Ochoa MC Minute L Rodriguez I Garasa S Perez-Ruiz E Inoges S. Antibody-dependent cell cytotoxicity: immunotherapy strategies enhancing effector NK cells. Immunol Cell Biol. (2017) 95:347–55. 10.1038/icb.2017.6 28138156

[69] Ferris RL Lenz HJ Trotta AM Garcia-Foncillas J Schulten J Audhuy F. Rationale for combination of therapeutic antibodies targeting tumor cells and immune checkpoint receptors: Harnessing innate and adaptive immunity through IgG1 isotype immune effector stimulation. Cancer Treat Rev. (2018) 63:48–60. 10.1016/j.ctrv.2017.11.008 29223828 PMC7505164

[70] Ang KK Berkey BA Tu X Zhang HZ Katz R Hammond EH. Impact of epidermal growth factor receptor expression on survival and pattern of relapse in patients with advanced head and neck carcinoma. Cancer Res. (2002) 62:7350–6. 12499279

[71] Bhat R Watzl C. Serial killing of tumor cells by human natural killer cells–enhancement by therapeutic antibodies. PLoS ONE (2007) 2:e326. 10.1371/journal.pone.0000326 17389917 PMC1828617

[72] Lee SC Srivastava RM Lopez-Albaitero A Ferrone S Ferris RL Natural killer (NK): dendritic cell (DC) cross talk induced by therapeutic monoclonal antibody triggers tumor antigen-specific T cell immunity Immunol Res. (2011) 50:248–54. 10.1007/s12026–011-8231–0 21717064 PMC3415245

[73] Trivedi S Srivastava RM Concha-Benavente F Ferrone S Garcia-Bates TM Li J. Anti-EGFR targeted monoclonal antibody isotype influences antitumor cellular immunity in head and neck cancer patients. Clin Cancer Res. (2016) 22:5229–37. 10.1158/1078–0432.ccr-15–2971 27217441 PMC5093040

[74] Pozzi C Cuomo A Spadoni I Magni E Silvola A Conte A. The EGFR-specific antibody cetuximab combined with chemotherapy triggers immunogenic cell death. Nat Med. (2016) 22 624–31. 10.1038/nm.4078 27135741

[75] Jie HB Srivastava RM Argiris A Bauman JE Kane LP Ferris RL. Increased PD-1(+) and TIM-3(+) TILs during cetuximab therapy inversely correlate with response in head and neck cancer patients. Cancer Immunol Res. (2017) 5:408–416. 10.1158/2326–6066.cir-16–0333 28408386 PMC5497750

[76] Jie HB Schuler PJ Lee SC Srivastava RM Argiris A Ferrone S. CTLA-4(+) Regulatory T Cells Increased in Cetuximab-Treated Head And Neck Cancer Patients Suppress NK Cell cytotoxicity and correlate with poor prognosis. Cancer Res. (2015) 75:2200–10. 10.1158/0008–5472.can-14–2788 25832655 PMC4452385

[77] Ghiringhelli F Menard C Terme M Flament C Taieb J Chaput N. CD4+CD25+ regulatory T cells inhibit natural killer cell functions in a transforming growth factor-beta-dependent manner. J Exp Med. (2005) 202:1075–85. 10.1084/jem.20051511 16230475 PMC2213209

[78] Chen A Ali N Boasberg P Ho AS. Clinical remission of cutaneous squamous cell carcinoma of the auricle with cetuximab and nivolumab. J Clin Med. (2018) 7:E10. 10.3390/jcm7010010 29320468 PMC5791018

[79] Dong ZY Zhang JT Liu SY Su J Zhang C Xie Z. EGFR mutation correlates with uninflamed phenotype and weak immunogenicity, causing impaired response to PD-1 blockade in non-small cell lung cancer. Oncoimmunology (2017) 6:e1356145. 10.1080/2162402x.2017.1356145 29147605 PMC5674946

[80] Hata A Katakami N Nanjo S Okuda C Kaji R Masago K. Programmed death-ligand 1 expression according to epidermal growth factor receptor mutation status in pretreated non-small cell lung cancer. Oncotarget (2017) 8:113807–16. 10.18632/oncotarget.22837 29371947 PMC5768364

[81] Ramjiawan RR Griffioen AW Duda DG. Anti-angiogenesis for cancer revisited: Is there a role for combinations with immunotherapy? Angiogenesis (2017) 20:185–204. 10.1007/s10456–017-9552-y 28361267 PMC5439974

[82] Liu XD Hoang A Zhou L Kalra S Yetil A Sun M. Resistance to antiangiogenic therapy is associated with an immunosuppressive tumor microenvironment in metastatic renal cell carcinoma. Cancer Immunol Res. (2015) 3:1017–29. 10.1158/2326–6066.cir-14–0244 26014097 PMC4561186

[83] Huang Y Yuan J Righi E Kamoun WS Ancukiewicz M Nezivar J. Vascular normalizing doses of antiangiogenic treatment reprogram the immunosuppressive tumor microenvironment and enhance immunotherapy. Proc Natl Acad Sci USA. (2012) 109:17561–6. 10.1073/pnas.1215397109 23045683 PMC3491458

[84] Huang Y Goel S Duda DG Fukumura D Jain RK. Vascular normalization as an emerging strategy to enhance cancer immunotherapy. Cancer Res. (2013) 73:2943–8. 10.1158/0008–5472.can-12–4354 23440426 PMC3655127

[85] Chen Y Ramjiawan RR Reiberger T Ng MR Hato T Huang Y. CXCR4 inhibition in tumor microenvironment facilitates anti-programmed death receptor-1 immunotherapy in sorafenib-treated hepatocellular carcinoma in mice. Hepatology (2015) 61:1591–602. 10.1002/hep.27665 25529917 PMC4406806

[86] Wallin JJ Bendell JC Funke R Sznol M Korski K Jones S. Atezolizumab in combination with bevacizumab enhances antigen-specific T-cell migration in metastatic renal cell carcinoma. Nat Commun. (2016) 7:12624. 10.1038/ncomms12624 27571927 PMC5013615

[87] Wei SC Levine JH Cogdill AP Zhao Y N.-A.Anang AS Andrews MC. distinct cellular mechanisms underlie Anti-CTLA-4 and Anti-PD-1 checkpoint blockade. Cell (2017) 170:1120–33.e17. 10.1016/j.cell.2017.07.024 28803728 PMC5591072

[88] Das R Verma R Sznol M Boddupalli CS Gettinger SN Kluger H Combination therapy with anti-CTLA4 and anti-PD1 leads to distinct immunologic changes *in-vivo*. J Immunol. (2015) 194:950–9. 10.4049/jimmunol.1401686 25539810 PMC4380504

[89] Postow MA Chesney J Pavlick AC Robert C Grossmann K Mcdermott D. Nivolumab and ipilimumab versus ipilimumab in untreated melanoma. N Engl J Med. (2015) 372:2006–17. 10.1056/NEJMoa1414428 25891304 PMC5744258

[90] Schwab KS Kristiansen G Schild HH S.Held EA Heine A Brossart P. Successful treatment of refractory squamous cell cancer of the head and neck with nivolumab and ipilimumab. Case Rep Oncol. (2018) 11:17–20. 10.1159/000485562 29515404 PMC5836192

[91] Liu JF Ma SR Mao L Bu LL Yu GT Li YC. T-cell immunoglobulin mucin 3 blockade drives an antitumor immune response in head and neck cancer. Mol Oncol. (2017) 11:235–47. 10.1002/1878–0261.12029 28102051 PMC5527458

[92] Koyama S Akbay EA Li YY Herter-Sprie GS Buczkowski KA Richards WG. Adaptive resistance to therapeutic PD-1 blockade is associated with upregulation of alternative immune checkpoints. Nat Commun. (2016) 7:10501. 10.1038/ncomms10501 26883990 PMC4757784

[93] Sakuishi K Apetoh L Sullivan JM Blazar BR Kuchroo VK Anderson AC. Targeting Tim-3 and PD-1 pathways to reverse T cell exhaustion and restore anti-tumor immunity. J Exp Med. (2010) 207:2187–94. 10.1084/jem.20100643 20819927 PMC2947065

[94] Lichtenegger FS Rothe M Schnorfeil FM Deiser K Krupka C Augsberger C. Targeting LAG-3 and PD-1 to Enhance T Cell activation by antigen-presenting cells. Front Immunol. (2018) 9:385. 10.3389/fimmu.2018.00385 29535740 PMC5835137

[95] Rotte A Jin JY Lemaire V. Mechanistic overview of immune checkpoints to support the rational design of their combinations in cancer immunotherapy. Ann Oncol. (2018) 29:71–83. 10.1093/annonc/mdx686 29069302

[96] Watts TH. TNF/TNFR family members in costimulation of T cell responses. Annu Rev Immunol. (2005) 23:23. 10.1146/annurev.immunol.23.021704.115839 15771565

[97] Deng WW Wu L Sun ZJ. Co-inhibitory immune checkpoints in head and neck squamous cell carcinoma. Oral Dis. (2018) 24:120–3. 10.1111/odi.12746 29480599

[98] Economopoulou P Kotsantis I Psyrri A. The promise of immunotherapy in head and neck squamous cell carcinoma: combinatorial immunotherapy approaches. ESMO Open (2016) 1:e000122. 10.1136/esmoopen-2016–000122 28848660 PMC5548974

[99] Bell RB Leidner RS Crittenden MR Curti BD Feng Z Montler R. OX40 signaling in head and neck squamous cell carcinoma: overcoming immunosuppression in the tumor microenvironment. Oral Oncol. (2016) 52:1–10. 10.1016/j.oraloncology.2015.11.009 26614363

[100] Guo Z Wang X Cheng D Xia Z Luan M Zhang S. PD-1 blockade and OX40 triggering synergistically protects against tumor growth in a murine model of ovarian cancer. PLoS ONE (2014) 9:e89350. 10.1371/journal.pone.0089350 24586709 PMC3937343

[101] Agematsu K Kobata T Sugita K Hirose T Schlossman SF Morimoto C Direct cellular communications between CD45R0 and CD45RA T cell subsets via CD27/CD70. J Immunol. (1995) 154:3627–35. 7706706

[102] Buchan S Manzo T Flutter B Rogel A Edwards N Zhang L OX40- and CD27-mediated co-stimulation synergize with anti-PD-L1 blockade by forcing exhausted CD8+ T cells to exit quiescence. J Immunol. (2014) 194:125–33. 10.4049/jimmunol.1401644 25404365 PMC4272895

[103] Buchan SL Fallatah M Thirdborough SM Taraban VY Rogel A Thomas LJ. PD-1 Blockade and CD27 Stimulation activate distinct transcriptional programs that synergize for CD8(+) T-Cell-driven antitumor immunity. Clin Cancer Res. (2018) 24:2383–94. 10.1158/1078–0432.ccr-17–3057 29514845 PMC5959006

[104] Wei H Zhao L Li W Fan K Qian W Hou S. Combinatorial PD-1 blockade and CD137 activation has therapeutic efficacy in murine cancer models and synergizes with cisplatin. PLoS ONE (2013) 8:e84927. 10.1371/journal.pone.0084927 24367702 PMC3868659

[105] Azpilikueta A Agorreta J Labiano S Perez-Gracia JL Sanchez-Paulete AR Aznar MA. Successful immunotherapy against a transplantable mouse squamous lung carcinoma with Anti-PD-1 and Anti-CD137 monoclonal antibodies. J Thorac Oncol. (2016) 11:524–36. 10.1016/j.jtho.2016.01.013 26845193

[106] Tolcher AW Sznol M Hu-Lieskovan S Papadopoulos KP Patnaik A Rasco DW. Phase Ib Study of Utomilumab (PF-05082566), a 4–1BB/CD137 agonist, in combination with pembrolizumab (MK-3475) in patients with advanced solid tumors. Clin Cancer Res. (2017) 23:5349–57. 10.1158/1078–0432.ccr-17–1243 28634283

[107] Prendergast GC Immune escape as a fundamental trait of cancer: focus on ID. Oncogene (2008) 27:3889–900. 10.1038/onc.2008.35 18317452

[108] Foy JP Bertolus C Michallet MC Deneuve S Incitti R Bendriss-Vermare N. The immune microenvironment of HPV-negative oral squamous cell carcinoma from never-smokers and never-drinkers patients suggests higher clinical benefit of IDO1 and PD1/PD-L1 blockade. Ann Oncol. (2017) 28:1934–41. 10.1093/annonc/mdx210 28460011

[109] Toulmonde M Penel N Adam J Chevreau C Blay JY Le Cesne A. Use of PD-1 targeting, macrophage infiltration, and ido pathway activation in sarcomas: a phase 2 clinical trial. JAMA Oncol. (2018) 4:93–97. 10.1001/jamaoncol.2017.1617 28662235 PMC5833654

[110] Zhang X Wang C Wang J Hu Q Langworthy B Ye Y. PD-1 blockade cellular vesicles for cancer immunotherapy. Adv Mater. (2018) 30:e1707112. 10.1002/adma.201707112 29656492

[111] Wang C Dickie J Sutavani RV Pointer C Thomas GJ Savelyeva N. Targeting head and neck cancer by vaccination. Front Immunol. (2018) 9:830. 10.3389/fimmu.2018.00830 29740440 PMC5924779

[112] Rothschild U Muller L Lechner A Schlosser HA Beutner D Laubli H. Immunotherapy in head and neck cancer - scientific rationale, current treatment options and future directions. Swiss Med Wkly (2018) 148:w14625. 10.4414/smw.2018.14625 29756633

[113] Fukuhara H Ino Y Todo T. Oncolytic virus therapy: a new era of cancer treatment at dawn. Cancer Sci. (2016) 107:1373–9. 10.1111/cas.13027 27486853 PMC5084676

[114] Liu BL Robinson M Han ZQ Branston RH English C Reay P. ICP34.5 deleted herpes simplex virus with enhanced oncolytic, immune stimulating, and anti-tumour properties. Gene Ther. (2003) 10:292. 10.1038/sj.gt.3301885 12595888

[115] Ribas A Dummer R Puzanov I VanderWalde A Andtbacka HI Michielin O. Oncolytic virotherapy promotes intratumoral T Cell Infiltration and Improves Anti-PD-1 Immunotherapy. Cell (2017) 170:1109–19.e10. 10.1016/j.cell.2017.08.027 28886381 PMC8034392

[116] Outh-Gauer S Alt M Le Tourneau C Augustin J Broudin C Gasne C. Immunotherapy in head and neck cancers: a new challenge for immunologists, pathologists and clinicians. Cancer Treatment Rev. (2018) 65:54–64 10.1016/j.ctrv.2018.02.008 29547766

[117] Lawrence MS Stojanov P Polak P Kryukov GV Cibulskis K Sivachenko A. Mutational heterogeneity in cancer and the search for new cancer-associated genes. Nature (2013) 499 214–8. 10.1038/nature12213 23770567 PMC3919509

[118] Outh-Gauer S Le Tourneau C Broudin C Scotte F Roussel H Hans S. Current events in immunotherapy for upper aerodigestive tract cancer. Ann pathol. (2017) 37:79–89. 10.1016/j.annpat.2016.12.013 28111039

[119] Partlova S Boucek J Kloudova K Lukesova E Zabrodsky M Grega M. Distinct patterns of intratumoral immune cell infiltrates in patients with HPV-associated compared to non-virally induced head and neck squamous cell carcinoma. Oncoimmunology (2015) 4:e965570. 10.4161/21624011.2014.965570 25949860 PMC4368144

[120] Badoual C Hans S Merillon N Van Ryswick C Ravel P Benhamouda N. PD-1-expressing tumor-infiltrating T cells are a favorable prognostic biomarker in HPV-associated head and neck cancer. Cancer Res. (2013) 73:128–38. 10.1158/0008–5472.can-12–2606 23135914

[121] Rice AE Latchman YE Balint JP Lee JH Gabitzsch ES Jones FR. An HPV-E6/E7 immunotherapy plus PD-1 checkpoint inhibition results in tumor regression and reduction in PD-L1 expression. Cancer gene therapy (2015) 22:454–62 10.1038/cgt.2015.40 26337747

[122] Massarelli E William W Johnson F Kies M Ferrarotto R Guo M. Combining immune checkpoint blockade and tumor-specific vaccine for patients with incurable human papillomavirus 16-related cancer: a phase 2 clinical trial. JAMA Oncol. (2018) 10.1001/jamaoncol.2018.4051. [Epub ahead of print]. 30267032 PMC6439768

[123] Kang H Kiess A Chung CH. Emerging biomarkers in head and neck cancer in the era of genomics. Nat Rev. (2015) 12:11–26 10.1038/nrclinonc.2014.192 25403939

[124] Hoesli RC Ludwig ML Michmerhuizen NL Rosko AJ Spector ME Brenner JC. Genomic sequencing and precision medicine in head and neck cancers. Eur J Surg Oncol. (2017) 43:884–92. 10.1016/j.ejso.2016.12.002 28034498 PMC5393934

[125] zur Hausen H. Papillomaviruses and cancer: from basic studies to clinical application. Nat Rev Cancer (2002) 2:342 10.1038/nrc798 12044010

[126] Feldman R Gatalica Z Knezetic J Reddy S Nathan CA Javadi N. Molecular profiling of head and neck squamous cell carcinoma. Head Neck (2016) 38:(Suppl. 1):E1625–38. 10.1002/hed.24290 26614708 PMC5063170

[127] Ritprajak P Azuma M. Intrinsic and extrinsic control of expression of the immunoregulatory molecule PD-L1 in epithelial cells and squamous cell carcinoma. Oral Oncol. (2015) 51:221–8. 10.1016/j.oraloncology.2014.11.014 25500094

[128] Moore EC Sun L Clavijo PE Friedman J Harford JB Saleh AD. Nanocomplex-based TP53 gene therapy promotes anti-tumor immunity through TP53- and STING-dependent mechanisms. Oncoimmunology (2018) 7:e1404216. 10.1080/2162402x.2017.1404216 29900037 PMC5993490

[129] Chung V Kos FJ Hardwick N Yuan Y Chao J Li D. Evaluation of safety and efficacy of p53MVA vaccine combined with pembrolizumab in patients with advanced solid cancers. Clin Trans Oncol. (2018) 10.1007/s12094-018-1932-2. [Epub ahead of print]. 30094792 PMC8802616

[130] Costa R Carneiro BA Agulnik M Rademaker AW Pai SG Villaflor VM. Toxicity profile of approved anti-PD-1 monoclonal antibodies in solid tumors: a systematic review and meta-analysis of randomized clinical trials. Oncotarget (2017) 8:8910–20. 10.18632/oncotarget.13315 27852042 PMC5352453

[131] Moslehi JJ Salem JE Sosman JA Lebrun-Vignes B Johnson DB. Increased reporting of fatal immune checkpoint inhibitor-associated myocarditis. Lancet (2018) 391:933. 10.1016/s0140–6736(18)30533–6 29536852 PMC6668330

[132] Chiang CS Lin YJ Lee R Lai YH Cheng HW Hsieh CH. Combination of fucoidan-based magnetic nanoparticles and immunomodulators enhances tumour-localized immunotherapy. Nat Nanotechnol (2018) 13:746–54. 10.1038/s41565–018-0146–7 29760523

[133] Wang C Wang J Zhang X Yu S Wen D Hu Q. *In situ* formed reactive oxygen species-responsive scaffold with gemcitabine and checkpoint inhibitor for combination therapy. Sci Transl Med. (2018) 10:eaan3682. 10.1126/scitranslmed.aan3682 29467299

